# Dynein links engulfment and execution of apoptosis via CED-4/Apaf1 in *C. elegans*

**DOI:** 10.1038/s41419-018-1067-y

**Published:** 2018-09-27

**Authors:** Rikke Hindsgaul Harders, Tine Hørning Morthorst, Anna Dippel Lande, Marianne Overgaard Hesselager, Ole Aalund Mandrup, Emøke Bendixen, Allan Stensballe, Anders Olsen

**Affiliations:** 1 Department of Chemistry and Biosciences, Aalborg University, Fredrik Bajers Vej 7H, Aalborg, DK-9220 Denmark; 2 Department of Molecular Biology and Genetics, Aarhus University, Gustav Wieds Vej 10C, Aarhus C, DK-8000 Denmark; 3 Department of Engineering, Aarhus University, Gustav Wieds Vej 10C, Aarhus C, DK-8000 Denmark; 4 Department of Health Science and Technology, Aalborg University, Fredrik Bajers Vej 7E, Aalborg, DK-9220 Denmark

## Abstract

Apoptosis ensures removal of damaged cells and helps shape organs during development by removing excessive cells. To prevent the intracellular content of the apoptotic cells causing damage to surrounding cells, apoptotic cells are quickly cleared by engulfment. Tight regulation of apoptosis and engulfment is needed to prevent several pathologies such as cancer, neurodegenerative and autoimmune diseases. There is increasing evidence that the engulfment machinery can regulate the execution of apoptosis. However, the underlying molecular mechanisms are poorly understood. We show that dynein mediates cell non-autonomous cross-talk between the engulfment and apoptotic programs in the *Caenorhabditis elegans* germline. Dynein is an ATP-powered microtubule-based molecular motor, built from several subunits. Dynein has many diverse functions including transport of cargo around the cell. We show that both dynein light chain 1 (DLC-1) and dynein heavy chain 1 (DHC-1) localize to the nuclear membrane inside apoptotic germ cells in *C. elegans*. Strikingly, lack of either DLC-1 or DHC-1 at the nuclear membrane inhibits physiological apoptosis specifically in mutants defective in engulfment. This suggests that a cell fate determining dialogue takes place between engulfing somatic sheath cells and apoptotic germ cells. The underlying mechanism involves the core apoptotic protein CED-4/Apaf1, as we find that DLC-1 and the engulfment protein CED-6/GULP are required for the localization of CED-4 to the nuclear membrane of germ cells. A better understanding of the communication between the engulfment machinery and the apoptotic program is essential for identifying novel therapeutic targets in diseases caused by inappropriate engulfment or apoptosis.

## Introduction

Dysregulation of apoptosis and engulfment contributes to the etiology of pathologies such as cancer, neurodegenerative and autoimmune diseases^1–6^. Several conserved core proteins involved in the execution of apoptosis were discovered in the nematode *Caenorhabditis elegans* where three types of apoptosis occur^7,8^. During embryogenesis and larval development, 131 cells undergo apoptosis (developmental)^9,10^. In the germline, around half of the germ cells are eliminated by apoptosis during adulthood (physiological)^11^. Finally, germ cells can undergo apoptosis if exposed to different stressors, DNA damage or checkpoint failure (damage-induced)^12^. The core apoptotic machinery is indispensable for all types of apoptosis and is comprised of the anti-apoptotic CED-9 (Bcl-2)^13^, the caspase CED-3 (caspase-9)^14^, and the pro-apoptotic adaptor CED-4 (Apaf1)^15^. Pro-apoptotic signals inhibit CED-9, which releases its inhibition of CED-4, which in turn activates CED-3^12,16–18^. In the germline, CED-4 localizes primarily to the nuclear membrane^19^, but mitochondrial localization has also been reported^20^. Upon apoptotic stimuli, such as DNA damage, CED-4 accumulation increases at the nuclear membrane^20,21^. Apoptosis culminates in engulfment and degradation of the apoptotic cell. In *C. elegans*, dead cells are engulfed and degraded by neighboring cells, such as the sheath cells in the germline^22^. Two partially redundant pathways are the main regulators of engulfment, the *ced-1*, *ced-6* and *ced-7* pathway and the *ced-2, ced-5, ced-12* and *ced-10* pathway^23^. The engulfment pathways cooperate with the apoptotic machinery to induce developmental apoptosis, since overactivation of engulfment causes more cells to die^24^, while a defect in engulfment causes more cells to survive under limited caspase activity^25–29^. However, little is known about the interaction between engulfment and the apoptotic machinery.

The dynein complex is a highly conserved microtubule-based motor of 1600 kDa that consists of two heavy chains, two intermediate chains, three different light chains, and two light intermediate chains^30^. Dynein has a variety of functions inside the cell; many related to transport or cell division^30^. In the germline of *C. elegans*, the heavy chain (DHC-1) shows an even cytoplasmic distribution and a strong accumulation at the nuclear membrane of all germ cells^31^. *C. elegans* dynein light chain 1 (DLC-1) has 95% sequence homology to the human light chains DYNLL1 and DYNLL2. Loss-of-function mutations of *dhc-1* and *dlc-1* cause embryonic lethality^32–34^. In *C. elegans*, DLC-1 is involved in nuclear migration^35^, regulation of METT-10 in germ cell differentiation^36^, and has a cell  non-autonomous anti-apoptotic function^37^. In this study, we show that germline-specific inactivation of *dlc-1* suppresses accumulation of apoptotic corpses in engulfment mutants. We show that both DLC-1 and DHC-1 localize to the nuclear membrane of apoptotic germ cells and that lack of either inhibits physiological apoptosis in mutants defective in engulfment. Furthermore, DLC-1 is required for accumulation of CED-4 at the nuclear membrane of germ cells, which is necessary for execution of apoptosis. Our study demonstrates a novel role of the dynein complex as a switch controlling live/death decisions in the germline in cooperation with the engulfment machinery.

## Results

### DLC-1 accumulates at the nuclear membrane of apoptotic germ cells

**Fig. 1:**
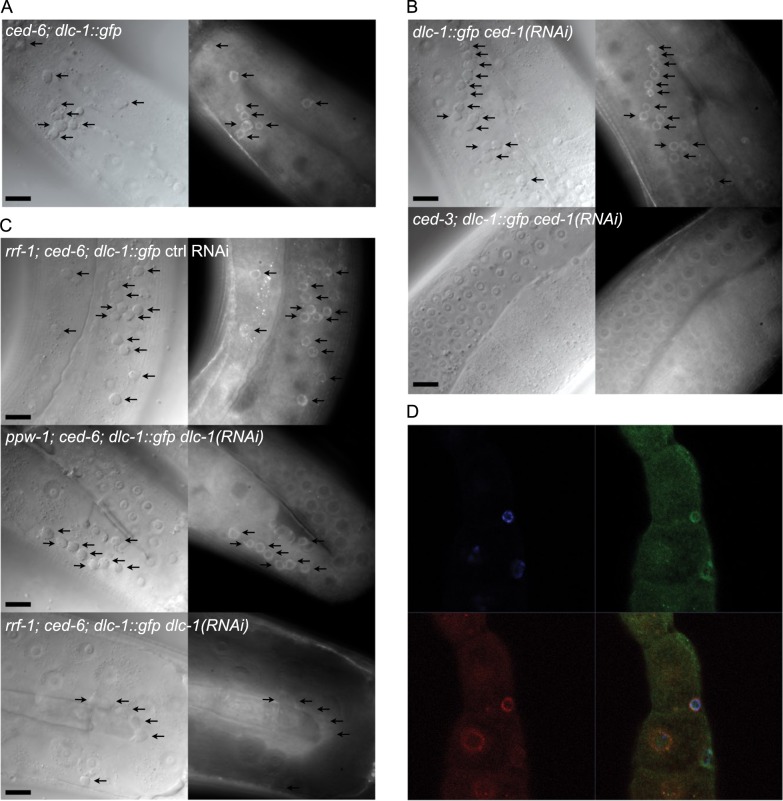
DLC-1 marks apoptotic germ cells. **a** DLC-1::GFP localizes to apoptotic germ cells (black arrows) in *ced-6(tm1826)* mutants. The apoptotic germ cells can be seen as button-like structures on the corresponding DIC image. **b** DLC-1::GFP localizes to apoptotic germ cells (black arrows) in animals treated with RNAi against *ced-1* (top). Mutation of *ced-3(n717)* completely abolishes the accumulation of apoptotic cells due to RNAi against *ced-1* (bottom). Blocking apoptosis removes the DLC-1::GFP rings. **c** Inactivation of *dlc-1* in somatic tissues (*ppw-1(pk2505)* mutant background) does not affect DLC-1::GFP localization to apoptotic cells. Inactivation of *dlc-1* in germ cells (*rrf-1(ok589)* mutant background) completely removes DLC-1::GFP localized to apoptotic cells. **d** DLC-1::GFP co-localizes with Mab414 antibody against the nuclear pore complex at the nuclear membrane (red) in apoptotic germ cells. DAPI (blue). Black scale bars: 10 µm

DLC-1 regulates germ cell apoptosis in response to ionizing radiation via a signal from somatic tissues^37^. However, DLC-1 is also expressed in the germline^36–38^. To establish if DLC-1 plays a role within germ cells undergoing apoptosis we expressed DLC-1::GFP in *ced-6(tm1826)* mutants, where apoptotic cells accumulate due to impaired engulfment^11^. Expression of the DLC-1::GFP transgene did not alter germline morphology and the fusion protein was found evenly distributed in healthy germ cells consistent with previous studies^38^. Strikingly, we found that DLC-1::GFP strongly localized to DIC positive apoptotic germ cells (Fig. 1a). To verify that the cells marked by DLC-1::GFP were indeed apoptotic, we expressed DLC-1::GFP in *ced-3(n717)* mutants, in which apoptosis is blocked^7^ and treated them with RNAi against *ced-1* to block engulfment. In *ced-3* mutants, we found no DIC positive corpses nor any germ cells marked with DLC-1::GFP, demonstrating that DLC-1::GFP accumulation specifically marks apoptotic cells (Fig. 1b). Physiological apoptosis also occurs in wild-type animals but fewer corpses are found because they are rapidly removed by engulfment. We found that some apoptotic cells in wild-type worms were also marked by the accumulation of DLC-1::GFP but generally to a lesser extent than in engulfment mutants (Fig. S1A). The expression of the single copy DLC-1::GFP transgene did not significantly alter the level of apoptosis in wild-type animals or engulfment mutants (Fig. S1B-D). The low number of apoptotic corpses in wild-type animals could perhaps mask an effect. Hence, we investigated the effect of the DLC-1::GFP transgene in *gla-3(ok2684)* mutants and worms treated with RNAi against the cytoplasmic polyadenylation-element-binding-protein-1 (CPEB1) homolog *cbp-3* as these have elevated apoptosis but normal engulfment^37,39^. No effect of the DLC-1::GFP transgene was observed in these (Fig. S1E and F). Thus, the extra copy of DLC-1::GFP does not influence germline apoptosis.

To establish if DLC-1::GFP is localized within the apoptotic germ cells or in the engulfing sheath cells, we took advantage of *rrf-1* and *ppw-1* mutants, where RNAi only works in the germline and somatic cells, respectively^40,41^. All experiments were performed in a *ced-6(tm1826)*; *dlc-1::gfp* background. In *ppw-1* animals treated with *dlc-1* RNAi, DLC-1::GFP clearly marked apoptotic cells similar to control animals (Fig. 1c), whereas DLC-1::GFP was absent from apoptotic cells in *rrf-1* animals treated with RNAi against *dlc-1* (Fig. 1c). Thus, DLC-1::GFP is localized inside dying germ cells.

Due to the distribution of DLC-1::GFP, we hypothesized that it was localized to the nuclear membrane. To investigate the subcellular localization of DLC-1::GFP, dissected gonads from *ced-6(tm1826)*; *dlc-1::gfp* animals were stained with the nuclear pore specific antibody Mab414. We observed that DLC-1::GFP co-localized with the nuclear membrane of apoptotic germ cells (Fig. 1d and Fig. S2).

To address the generality of DLC-1::GFP localization to apoptotic germ cells, we investigated additional engulfment mutants. DLC-1::GFP also marked apoptotic germ cells in *ced-1(e1735)*, *ced-2(n1994)*, *ced-5(tm1950)*, *ced-7(n1996)*, and *ced-12(n3261)* mutants as well as partial loss of function *ced-9(n1653)* mutants that have elevated apoptosis but normal engulfment^11^ (Fig. S3). We also examined comma stage embryos but could not detect any DLC-1::GFP localization to somatic apoptotic  cells (Fig. S4).

### Depletion of *dlc-1* suppresses apoptosis in engulfment mutants

**Fig. 2:**
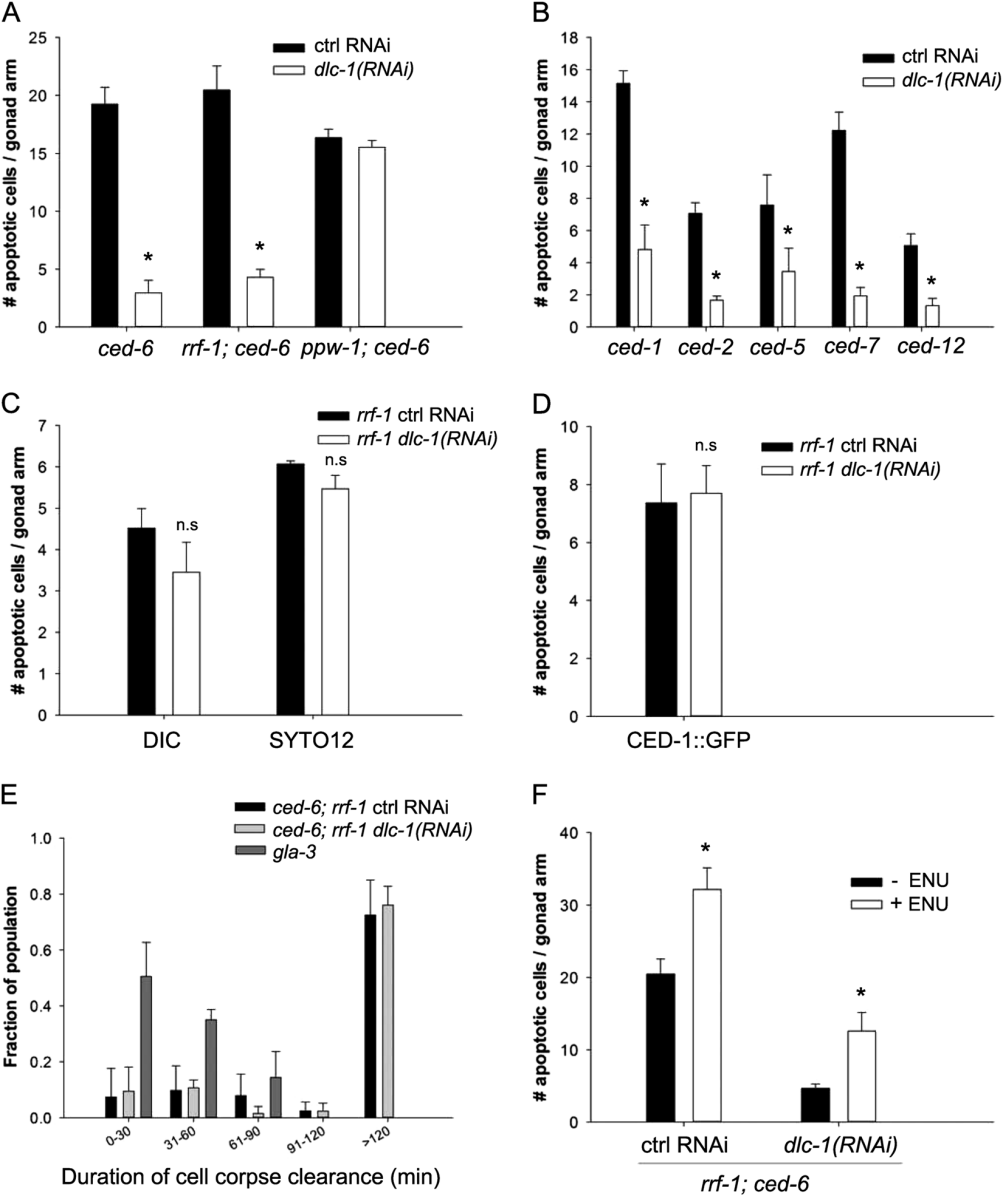
Lack of DLC-1 suppresses the accumulation of apoptotic germ cells in engulfment mutants. **a** RNAi against *dlc-1* significantly reduces the number of apoptotic germ cells in *ced-6(tm1826)* mutants when *dlc-1* is inactivated in germ cells (wt or *rrf-1(ok589)* mutant background). Inactivating *dlc-1* specifically in somatic tissues (*ppw-1(pk2505)* mutant background) has no effect on the number of apoptotic germ cells. Bars represent mean ± SD of three independent experiments. *p < 0.05. All strains contain the DLC-1::GFP transgene. **b** RNAi against *dlc-1* significantly reduces the number of apoptotic cells in engulfment mutants from the two major engulfment pathways. Bars represent mean ± SD of three independent experiments. *p < 0.05. All strains contain the DLC-1::GFP transgene. **c**, **d** RNAi against *dlc-1* specifically in germ cells does not affect the number of apoptotic germs cells in *rrf-1 (ok589)* animals that are not defective in engulfment. Bars represent mean ± SD of three independent experiments. **e** Time lapse experiment measuring the engulfment time for apoptotic germ cells. RNAi against *dlc-1* does not accelerate the clearance of apoptotic cells in *rrf-1 (ok589)*; *ced-6(tm1826)*; *dlc-1::gfp* mutants. *gla-3 (ok2684)* mutants have wild-type rate of engulfment. Bars represent mean ± SD of three independent experiments. **f** ENU can induce apoptosis in *rrf-1(ok589)*; *ced-6(tm1826)*; *dlc-1::gfp* mutants treated with RNAi against *dlc-1*. Bars represent mean ± SD of three independent experiments for each strain. *p < 0.05. *n.s*. not significant

To uncover the function of DLC-1 in the apoptotic cells, we treated *ced-6(tm1826)* mutants with RNAi against *dlc-1*. Depletion of DLC-1 did not cause dramatic changes to the germline morphology or the number of germ cells. However, lack of DLC-1 completely suppressed the accumulation of unengulfed apoptotic cells in *ced-6* mutants (Fig. 2a). In agreement with the tissue-specific localization of DLC-1 in germ cells, removal of DLC-1 only in germ cells (*rrf-1* mutants) was sufficient to suppress accumulation of unengulfed apoptotic cell corpses (Fig. 2a). Removal of DLC-1 only in somatic tissues (*ppw-1* mutants) had no effect (Fig. 2a). A similar reduction in the number of unengulfed apoptotic cell corpses was observed in *ced-1(e1735)*, *ced-2(n1994)*, *ced-5(tm1950)*, *ced-7(n1996)*, and *ced-12(n3261)* mutants following RNAi against *dlc-1* (Fig. 2b), thus the effect is not specific for *ced-6* mutants.

To investigate whether DLC-1 also affects apoptosis in animals with normal engulfment, we treated *rrf-1(ok589)* mutants with RNAi against *dlc-1*. Using DIC microscopy, we did not observe any effect of *dlc-1* RNAi on apoptosis (Fig. 2c). This could be due to the low number of apoptotic cells in wild-type animals. We therefore used SYTO12 and CED-1::GFP to quantify apoptotic cells, as these methods generally visualize more apoptotic cells than DIC. However, *dlc-1* RNAi still did not influence apoptosis (Fig. 2c, d). This suggests that lack of *dlc-1* specifically affects apoptosis or engulfment in mutants defective in engulfment. The reduction in corpse numbers due to lack of DLC-1 could be the result of faster removal of apoptotic cells. To test this we measured engulfment rate of apoptotic cells in *rrf-1(ok589)*; *ced-6(tm1826)*; *dlc-1::gfp* mutants after *dlc-1* RNAi. We included *gla-3(ok2684)* mutants as positive controls representing wild-type engulfment rates^42^. We did not observe any difference in engulfment rate due to *dlc-1* depletion (Fig. 2e), indicating that DLC-1 does not affect engulfment rate.

Next, we tested whether the reduction in apoptotic cells could be due to an impaired ability of the germ cells to initiate apoptosis. We treated *rrf-1(ok589)*; *ced-6(tm1826)* animals with the alkylating agent N-ethyl-N-nitrosourea (ENU), which induces apoptosis in *C. elegans*^43^. Both in control animals and in animals treated with RNAi against *dlc-1*, ENU significantly increased germ cell apoptosis (Fig. 2f) showing that the core apoptotic machinery can still be activated. Consistent with this, exposure to UV also induced apoptosis in both controls and *dlc-1(RNAi)* worms (Fig. S5). Taken together, our data suggest that lack of DLC-1 specifically inhibits physiological apoptosis when engulfment is defective.

### DHC-1 localizes to normal and apoptotic germ cells

Since DLC-1 is a part of cytoplasmic dynein we next investigated whether the dynein heavy chain, DHC-1, also marks apoptotic cells. We observed that expression of DHC-1::GFP did not influence the morphology of the germline and that DHC-1::GFP localized to the nuclear membrane of healthy germ cells in wild-type animals (Fig. S6) in agreement with previous studies^31^.

**Fig. 3:**
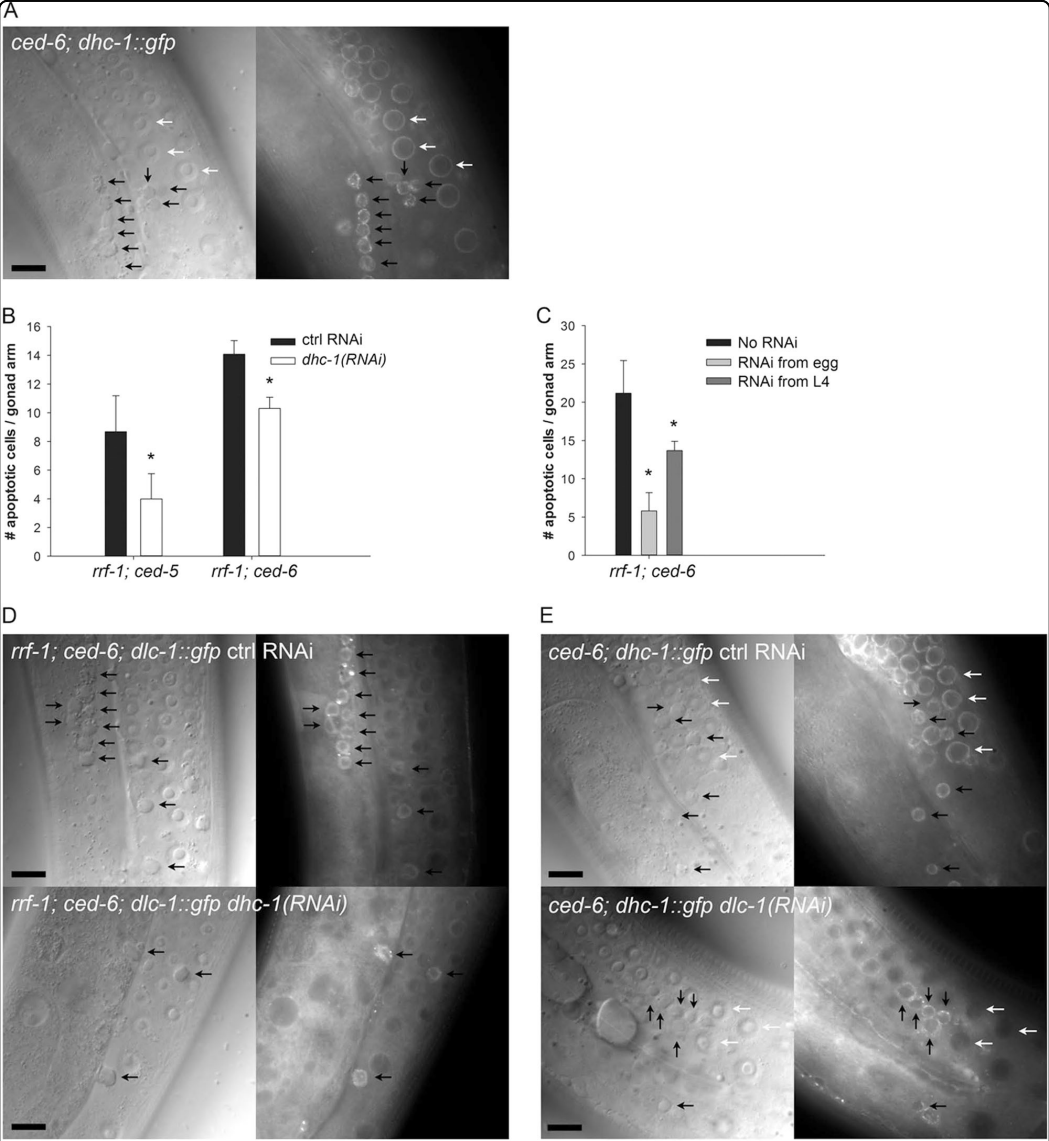
DHC-1 localizes to apoptotic germ cells and affects the execution of apoptosis in engulfment mutants. **a** DHC-1::GFP marks both normal (white arrows) and apoptotic germ cells (black arrows) in *ced-6(tm1826)* mutants. **b** RNAi against *dhc-1* from the L4 stage specifically in germ cells reduces the number of apoptotic germ cells in *rrf-1(ok589)*; *ced-6(tm1826)*; *dlc-1::gfp* and *rrf-1(ok589)*; *ced-5(tm1950)*; *dlc-1::gfp* mutants. Bars represent mean ± SD of three independent experiments. *p < 0.05. **c** RNAi against *dhc-1* from egg gives a bigger reduction in the number of apoptotic cells. Bars represent mean ± SD of three independent experiments. *p < 0.05. **d** RNAi inactivation of *dhc-1* does not alter the localization of DLC-1::GFP to cell corpses with 94% (15/16) being positive compared to 93% (50/54) in controls. Representative pictures are shown with corpses marked (black arrows). **e** RNAi against *dlc-1* removes DHC-1::GFP from healthy germ cells with 14% (32/222) being positive compared to 100% (208/209) in controls. RNAi against *dlc-1* does not remove DHC-1::GFP from apoptotic cells with 88% (74/84) being positive compared to 98% (59/60) in controls. Representative pictures are shown with corpses and healthy germ cells marked with black and white arrows, respectively. Black scale bars: 10 µm

In *ced-6* mutants, DHC-1::GFP was also found on the nuclear membrane of healthy germ cells (Fig. 3a, white arrows), but we also made the novel observation that DHC-1::GFP accumulates at the nuclear membrane of DIC positive apoptotic germ cells (Fig. 3a, black arrows). No change in germline apoptosis due to the expression of DHC-1::GFP was seen (Fig. S6B). Next, we investigated whether inactivation of *dhc-1* would suppress apoptosis in engulfment defective mutants similar to *dlc-1*. We observed that RNAi against *dhc-1* from egg causes larval arrest. RNAi against *dhc-1* from the L4 larval stage allowed for development into fertile adults and resulted in a significant reduction in the number of apoptotic cells in both *ced-5(tm1950)* and *ced-6(tm1826)* mutants (Fig. 3b). RNAi against *dhc-1* from egg only in the germline (*rrf-1(ok589)* background) does not cause larval arrest and only mildly affects germline morphology and germ cell number. In these animals, an even stronger reduction of apoptotic cells was observed comparable to RNAi against *dlc-1* (Fig. 3c). No significant effect was observed following RNAi against *dhc-1* in a *rrf-1(ok589)* mutant background with wild-type engulfment (Fig. S6C). These observations support a model where both DLC-1 and DHC-1 regulate germ cell apoptosis when engulfment is defective.

### Dynein regulators are required for correct DHC-1 localization

**Fig. 4:**
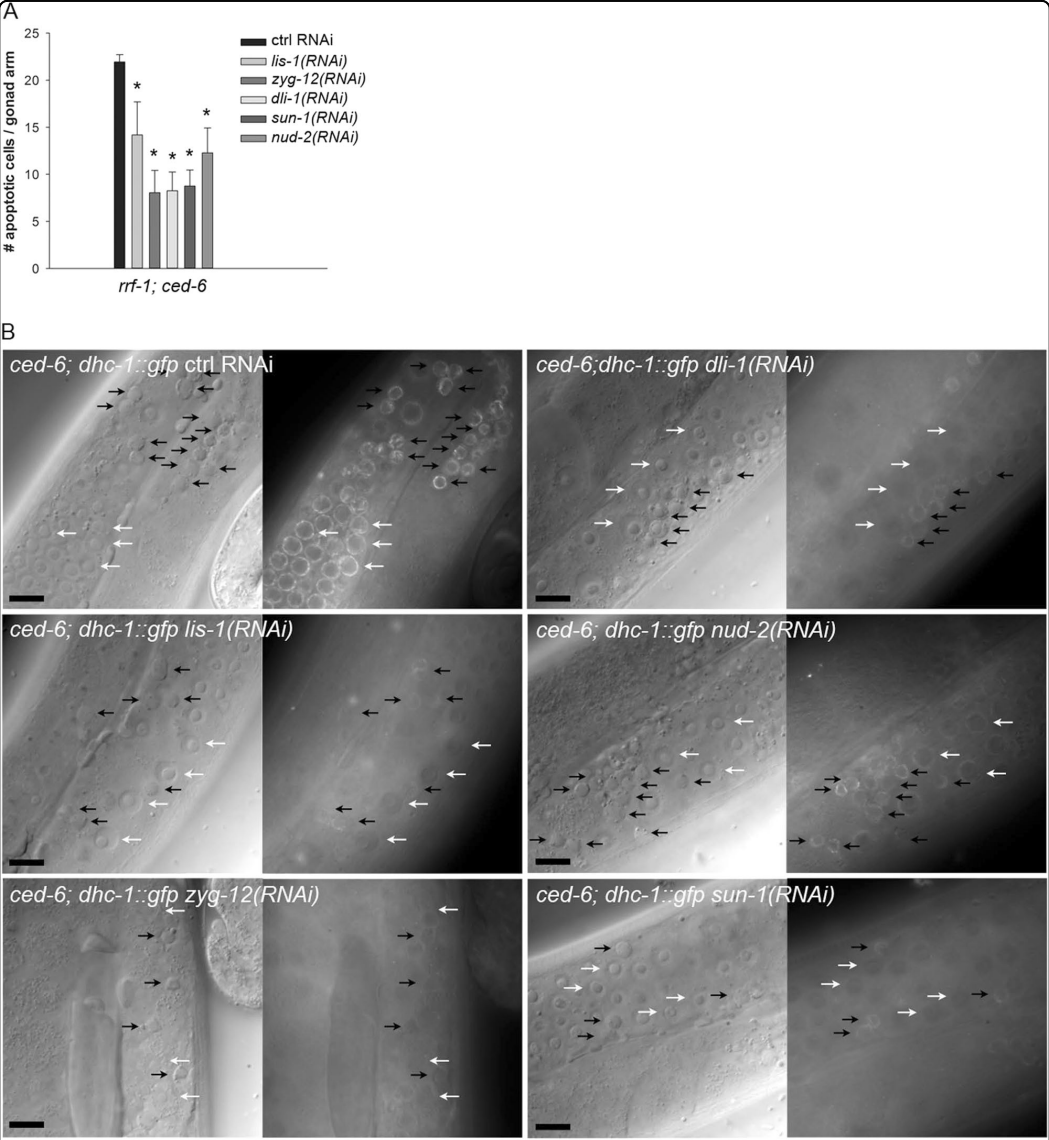
Dynein regulators affect apoptosis and DHC-1 localization. **a** RNAi inactivation of genes known to influence dynein function and localization leads to a significant reduction of apoptotic corpses in *rrf-1(ok589)*; *ced-6(tm1826)*; *dlc-1::gfp* mutants. Bars represent mean ± SD of three independent experiments. *p < 0.05. **b** RNAi against *dli-1* (0/133), *lis-1* (0/123), *zyg-12* (0/70), and *sun-1* (1/24) abolishes perinuclear accumulation of DHC-1::GFP in healthy germ cells. RNAi against *nud-2* significantly reduces the level of DHC-1::GFP (Fig. S7A) but does not remove it completely (172/179). In apoptotic germ cells, the level of DHC-1::GFP is significantly reduced by RNAi against *dli-1* (22/24), *lis-1* (40/61), *sun-1* (11/13), and *nud-2* (49/86) and nearly abolished by RNAi against *zyg-12* (1/20) (Fig. S7B). Numbers in brackets denote (GFP positive cells/total). Representative pictures are shown with corpses and healthy germ cells marked with black and white arrows, respectively. Black scale bars: 10 µm

A regulatory dependency between DLC-1 and DHC-1 exists^32^. We thus investigated whether DLC-1 and DHC-1 influenced the localization of each other. Inactivation of *dhc-1* in *ced-6(tm1826)*; *dlc-1::gfp* mutants did not alter the localization of DLC-1::GFP to apoptotic germ cells (Fig. 3d). Inactivation of *dlc-1* on the other hand removed the perinuclear localization of DHC-1::GFP in healthy germ cells in *ced-6(tm1826)* mutants (Fig. 3e, white arrows). Interestingly, inactivation of *dlc-1* only slightly reduced the fraction of DHC-1::GFP positive apoptotic cells (Fig. 3e, black arrows). This suggests that in healthy germ cells, DHC-1 binds to a perinuclear anchor in a DLC-1 dependent manner. In apoptotic cells, the DHC-1 binding dynamics could be different since DHC-1 is not completely removed from the nuclear membrane in the absence of DLC-1. We speculate that additional DHC-1 binding anchors could be present in apoptotic corpses. To further investigate this, we turned to proteins known to influence dynein. Dynein light intermediate chain 1, DLI-1, anchors DHC-1 to the nuclear membrane^31^. ZYG-12 (*zyg*ote defective) is a member of the hook family of linker proteins and is required for nuclear membrane localization of dynein^44^. SUN-1 (matefin MTF-1) is a nuclear envelope protein involved in meiotic progression^45^ and binds to ZYG-12. LIS-1 (lissencephaly protein *Lis1)* is a dynein assistant found throughout the cytoplasm and at the cell cortex and nuclear periphery. LIS-1 is required for binding of dynein to microtubules^46^. NUD-2, homologous to NudE and NudEL, cooperates with LIS-1 as an effector of dynein^47–49^. Germline-specific RNAi against *dli-1, lis-1*, *zyg-12*, *sun-1*, and *nud-2* significantly suppressed the number of apoptotic germ cells in *rrf-1(ok589)*; *ced-6(tm1826)*; *dlc-1::gfp* mutants (Fig. 4a). This supports that the inhibition of apoptosis by RNAi against *dlc-1* and *dhc-1* is via a dynein-dependent mechanism. Furthermore, the perinuclear localization of DHC-1::GFP in healthy germ cells was absent following knockdown of *dli-1, lis-1*, *zyg-12*, and *sun-1*, and significantly reduced by *nud-2* RNAi (Fig. 4b, white arrows and Fig. S7A). DHC-1::GFP accumulation in apoptotic germ cells was greatly reduced (*dli-1, lis-1*, *sun-1*, and *nud-2* RNAi) or completely abolished (*zyg-12* RNAi) (Fig. 4b, black arrows and Fig. S7B). In agreement with the observation that lack of DHC-1 does not alter DLC-1 localization, the accumulation of DLC-1::GFP in apoptotic cells was not disrupted by RNAi against any of these genes (Fig. S7C). Thus, known regulators of dynein affect DHC-1 localization in healthy and apoptotic germ cells, but they do not affect DLC-1 localization, demonstrating that despite their similar effect on apoptosis, DLC-1 and DHC-1 are recruited to apoptotic cells by different mechanisms.

### DLC-1 is required for localization of CED-4 to the nuclear membrane

**Fig. 5:**
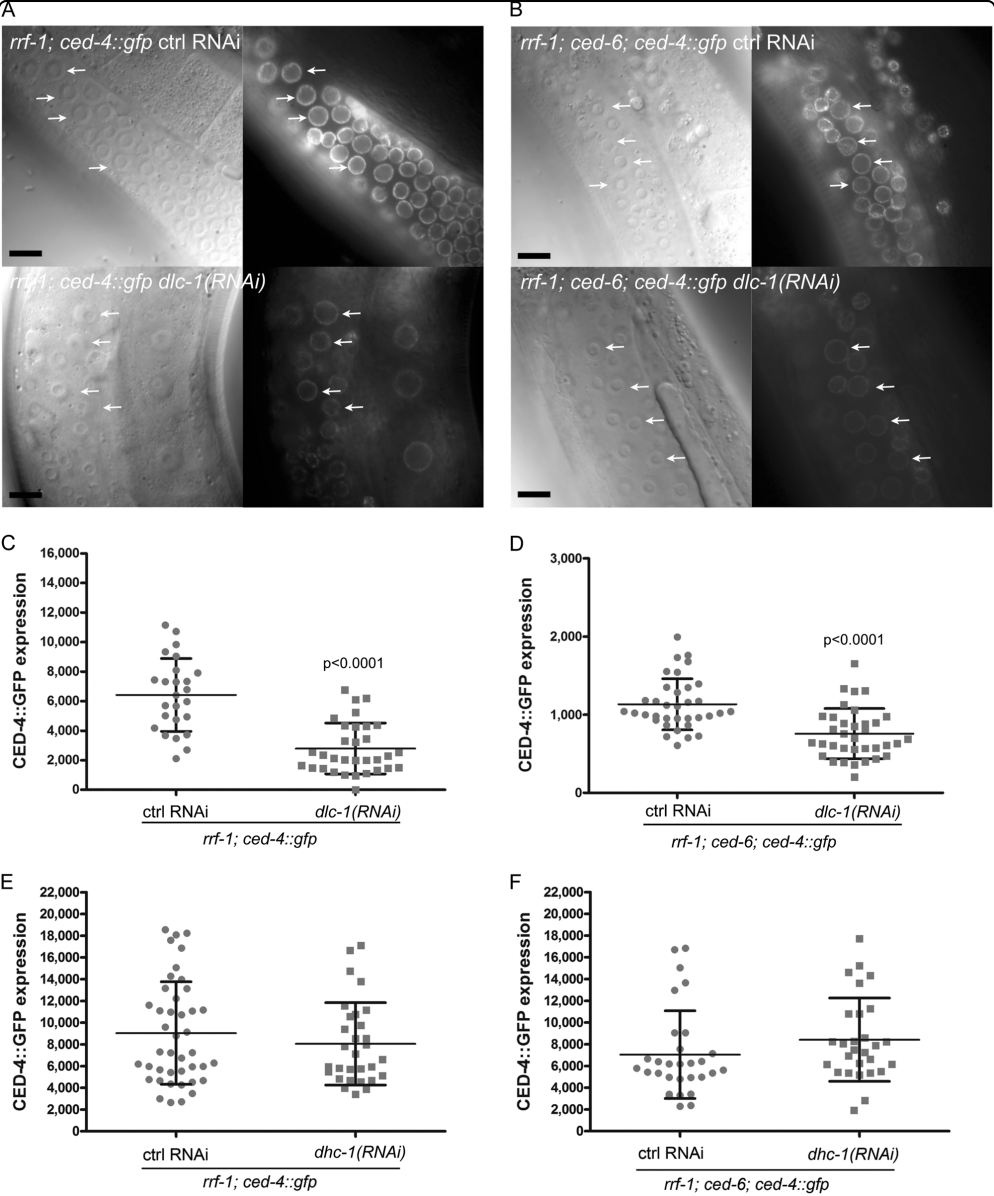
DLC-1 regulates localization of CED-4 in germ cells. **a** Nuclear membrane localized CED-4::GFP in healthy germ cells (white arrows) is greatly reduced by germ cell specific (*rrf-1* mutant) RNAi against *dlc-1*. **b** RNAi against *dlc-1* reduces CED-4 localization to the nuclear membrane of healthy germ cells (white arrows) in *ced-6(tm1826)* mutants. **c**, **d** Quantification of CED-4::GFP localization at the nuclear membrane of germ cells with or without RNAi against *dlc-1*. Graphs show one representative experiment of three independent replicates. Bars represent mean ± SD. **e**, **f** Quantification of CED-4::GFP localization at the nuclear membrane of germ cells with or without RNAi against *dhc-1*. Graphs show one representative experiment of two independent replicates. Bars represent mean ± SD. Black scale bars: 10 µm

The core apoptotic machinery protein CED-4 is localized at the nuclear membrane of healthy and apoptotic germ cells^19^, and is required for execution of apoptosis^11^. Since DLC-1 and DHC-1 both affect apoptosis and localize to the nuclear membrane of apoptotic cells, we investigated whether they change the localization of CED-4. We used a strain expressing CED-4::GFP under the *ced-4* promoter and crossed it into *rrf-1* and *ced-6* mutants. RNAi against *dlc-1* resulted in a significant reduction in CED-4::GFP accumulation at the nuclear membrane of healthy germ cells in *rrf-1* (Fig. 5a, c) and *ced-6; rrf-1* mutants (Fig. 5b, d). We tested whether DHC-1 could also regulate the localization of CED-4. However, RNAi against *dhc-1* did not affect CED-4 localization (Fig. 5e, f), and neither did RNAi against *dli-1, lis-1*, *zyg-12*, *sun-1*, and *nud-2* (data not shown). Interestingly, *ced-6* mutants in themselves had lower levels of CED-4::GFP in the germline (Fig. 5c–f), demonstrating that both CED-6 and DLC-1 regulates CED-4. This is in agreement with our observation that DHC-1 and its regulators do not affect DLC-1 localization and the notion that DLC-1 and DHC-1 induce apoptosis through partially independent mechanisms. The CED-4 protein sequence reveals two possible DLC-1 binding motifs. The first motif, ^169^ASQAL, resembles the identified DLC-1 binding site in the methyltransferase METT-10^36^. The second motif, ^301^AASQT, resembles the TASQT binding motif of African swine fever virus protein p54^50^. To test if DLC-1 interacts directly with CED-4, we co-immunoprecipitated DLC-1::GFP using GFP-trap® and probed for binding of CED-4 using an antibody against CED-4. We verified pull-down of DLC-1::GFP using an antibody against GFP. However, we could not detect CED-4 and thus not establish a direct interaction between DLC-1 and CED-4 (Fig. S8). The lack of CED-4 signal could be due to the sensitivity of the CED-4 antibody. Hence we performed the pull-down of DLC-1::GFP again and analyzed the bound proteins using mass spectrometry. Again, we were able to verify pull-down of DLC-1::GFP and an additional 48 proteins but we did not detect CED-4 (Table S1).

## Discussion

**Fig. 6:**
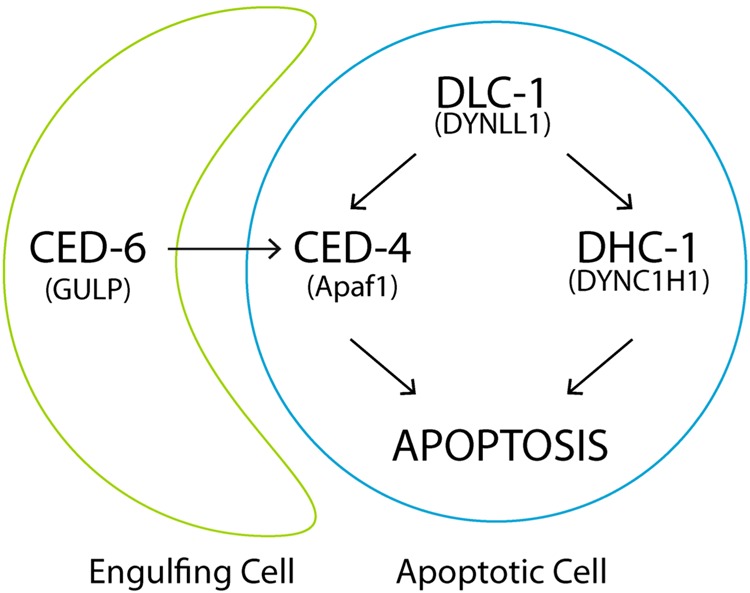
DLC-1 and engulfment regulate physiological apoptosis through parallel pathways. A model proposing pathways through which DLC-1, DHC-1 and engulfment regulates the induction of physiological apoptosis in the germline of *C. elegans*

Physiological apoptosis is a complex process that determines which of hundreds of germ cells are to be eliminated, however not much is known about the mechanisms that control this life/death decision. We demonstrate that dynein is one such switch and that it is working in an intricate pathway together with the engulfment machinery. We propose that the lack of *dlc-1* leads to diminished CED-4 accumulation on the nuclear membrane, which in turn inhibits the initiation of apoptosis (Fig. 6).

It has been shown that the engulfment pathways promote apoptosis. In animals with limited caspase activity, a defect in engulfment causes more cells to survive than in animals with normal engulfment^25,26^. Conversely, an injured cell can be induced to undergo apoptosis in mutants with overactivated engulfment^24^. Furthermore, for the death of the NSM sister cell, CED-1 was found to be required for the formation of a CED-3 gradient in the NSM precursor cell^28^. These studies have looked at somatic cells undergoing apoptosis during development. We propose that dynein is a pro-apoptotic protein complex regulating physiological apoptosis in cooperation with the engulfment machinery in the germline. Normally, when engulfment is inhibited, cells will continue to die due to the effective dynein switch. When dynein is compromised in this background, almost all physiological apoptosis is inhibited. In wild-type animals, RNAi against dynein has little effect due to the pro-apoptotic signal from the engulfment machinery overruling the inhibitory signal from lack of dynein, resulting in normal levels of physiological apoptosis (Fig. 6). At the molecular level, we propose that the dynein and engulfment switch converge on CED-4, as lack of CED-6 reduces the level of CED-4, and DLC-1 is required for correct CED-4 localization at the nuclear membrane. We propose that when either DLC-1 or engulfment is compromised, CED-4 levels on the nuclear membrane is reduced, however not enough to affect the induction of apoptosis. Only when both DLC-1 and engulfment are compromised, will the level of CED-4 on the nuclear membrane be too low to initiate apoptosis, thus blocking physiological apoptosis. This is in agreement with the previous studies showing that the pro-survival effect of reduced engulfment requires a limited caspase activity. In our case, this could be achieved by inactivation of DLC-1, which lowers the level of CED-4 at the nuclear membrane, thereby blocking the activation of the caspase CED-3. Since we observed that ENU could still induce apoptosis even when lack of *dlc-1* inhibited physiological apoptosis in engulfment defective mutants, DNA damage must regulate CED-4 through a different pathway than DLC-1 and engulfment to induce apoptosis.

How DLC-1 regulates CED-4 still needs to be investigated. Even though CED-4 contains two potential DLC-1 binding sites, we did not observe any direct interaction between the two. Therefore, at this point we cannot rule out a direct interaction between DLC-1 and CED-4. It is also possible that DLC-1 regulates CED-4 localization and expression by other means than a direct interaction. Beside dynein, DLC-1 (and homologues in various species) is known to interact with a large number of proteins^51^ and these could in principle all act as adaptors between DLC-1 and CED-4. Our DLC-1::GFP co-IP identified three additional candidates SAO-1(R10D12.14), RACK-1(K04D7.1), and DPF-2(C27C12.7).

Our data shows that DLC-1 is required for CED-4 localization to at least normal germ cells. Since RNAi against *dlc-1* inhibits apoptosis, this indicates that either (i) DLC-1 also regulates CED-4 localization to cells starting to die of apoptosis, or (ii) that the level of CED-4 on healthy germ cells is an indicator of whether they can undergo apoptosis or not. Previous studies have shown an intricate interplay between the regulation of different dynein subunits in both mammals and *C. elegans*^31,32,52^. Specifically, depletion of *dlc-1* can suppress the lethal phenotype of a conditional *dhc-1* loss of function allele, demonstrating a regulatory dependency between DLC-1 and DHC-1^32^. Our study strengthens this notion of a complex interplay between the two subunits, as we find that inactivation of *dlc-1* completely removes DHC-1 from the nuclear membrane of healthy germ cells but not from apoptotic cells. However, recruitment of DLC-1 to apoptotic cells proceeded normally in the absence of DHC-1, indicating that DLC-1 must be recruited independently of DHC-1 to apoptotic cells. This is supported by our observations that RNAi against the dynein-associated proteins DLI-1, LIS-1, ZYG-12, SUN-1, and NUD-2, all affect the localization of DHC-1 to healthy and dying germ cells, whereas none of these had any effect on the recruitment of DLC-1 to dying germ cells. Collectively, our data suggest that DLC-1 and DHC-1 are recruited to dying germ cells by two different mechanisms and that DLC-1 might be recruited prior to DHC-1 during apoptosis. Furthermore, we did not observe any effect on CED-4 localization when DHC-1 or the dynein effectors were inactivated, which is in agreement with the observation that they do not affect DLC-1 localization. Thus, they must promote apoptosis through a different but overlapping mechanism than DLC-1. DHC-1 functions as a molecular cargo transporter and is involved in a myriad of cellular processes including pronuclear migration and spindle positioning. Several of these functions could potentially be linked to apoptosis and explain our observations. For example, regulatory connections between cell cycle checkpoints and apoptosis have been established. Another possibility is that DHC-1 might bring other proteins required together with CED-4 to the nuclear membrane to initiate apoptosis, or be required for the correct orientation of DLC-1 or CED-4 to perform their actions.

Engulfment-mediated cell killing has also been observed in mammals. For example, macrophages promote the killing of developing neurons^53^ as well as stressed neurons^54^. Furthermore, phagocytosis also promotes the killing of tumor cells^55,56^ and elimination of macrophages rescues vascular endothelial cells destined to die in the rat eye^57^. It will be interesting to see if dynein also cooperates with phagocytosis to promote apoptosis in mammals.

## Materials and methods

If not otherwise stated all chemicals were purchased from Sigma-Aldrich, Copenhagen, Denmark.

### Strains and culture conditions

All strains were maintained at 20 °C on standard Nematode Growth Medium (NGM) plates spotted with *Escherichia coli* strain OP50. The following strains were used: Wild-type N2, linkage group I: CB3203 *ced-1(e1735)*, MT11068 *ced-12(n3261)*, RB798 *rrf-1(ok589)*, NL2550 *ppw-1(pk2505)*, RB2026 *gla-3(ok2684)*, RB1463 *cas-2(ok1676)*, XR6 *lagr-1(gk327)I;opIs219[ced-4p::ced-4::GFP unc-119(+)]*. Linkage group II: OLS400 *aarSi1[Pdlc-1::DLC-1::GFP cb-unc-119(+)]*. Linkage group III: *ced-6(tm1826)*, MT4983 *ced-7(n1996)*, MT3970 *ced-9(1653); mab-5(mu114)*. Linkage group IV: MT4952 *ced-2(n1994)*, MT1522 *ced-3(n717), ced-5(tm1950)*, CB1196 *unc-26(e1196)*, OD203 *(unc-119(ed3) III; orIs17 [Pdhc-1::GFP::dhc-1;unc-119(+)];Itls37 [pAA64; pie-1/mCherry::his-58; unc-119(+)] IV), VC592 pmk-1(ok811)* IV/nT1[qls51] (IV,V). Linkage group V: RB801 *hum-2(ok596)*, MD701 (*bcIs39*[P(*lim-7)ced-1*::GFP +*lin-15*(+)]).

For generation of double mutants, the presence of *aarSi1*, *orIs17, opIs219*, and *bcls39* in the F2 and F3 generation was scored by GFP expression. The presence of *ced-5, ced-6, hum-2*, and *rrf-1* was selected for by standard PCR. The presence of *ced-1, ced-2, ced-7*, and *ced-12* was selected for by the presence of unengulfed cell corpses in the embryos. Selection for *ced-3* and *ppw-1* was done by using the *unc-26(e1196)* and *cas-2(ok1676)* mutations as markers, respectively. *ced-9* was selected for by means of sterility at 25 °C. The *lagr-1(gk327)* mutation was crossed out of the RX6 strain by screening F2s with PCR.

### RNAi

The RNAi clones against *dlc-1* (T26A5.9), *zyg-12* (ZK546.1), *dli-1* (C39E9.14), *sun-1* (F57B1.2), *nud-2* (R11A5.2), and *cpb-3* (B0414.5) were from the OpenBiosystems RNAi Library. The RNAi clones against *dhc-1*(T21E12.4) and *lis-1*(T03F6.5T03F6.5) were from the Ahringer RNAi Library (Source BioScience LifeSciences, Nottingham, UK). RNAi was performed by feeding on NGM plates containing 1 mM isopropyl thiogalactoside and ampicillin (100 μg/ml) as described^58^. Worms fed HT115 bacteria containing an empty pL4440 vector (ctrl RNAi) were used as controls. All assays were performed with worms grown on RNAi for one generation, either from eggs or from L4 as indicated.

### Quantification of apoptosis

Cells undergoing apoptosis were distinguished from normal cells as refractile, button-like discs by DIC microscopy (Zeiss Axiophot Microscope equipped with an Andor Zyla 4.2 sCMOS camera) as described^59^. Alternatively, the CED-1::GFP (P_*lim-7*_*ced-1::*GFP, *bcIs39*) reporter was used to score apoptotic cells by fluorescence microscopy (Leica DMI3000B microscope equipped with an Olympus DP72 camera) as previously described^60^. Briefly, worms were synchronized as eggs and apoptosis was scored after 72 and 96 h. SYTO®12 staining of apoptotic corpses was performed on 4 days old worms by incubating them in 33 µM SYTO®12 green fluorescent nucleic acid stain (Invitrogen, Life Technologies, Naerum, Denmark) in S-basal for 3–4 h in the dark, rotating. The worms were transferred to fresh NGM or RNAi plates to recover for 30–60 min. For all procedures, worms were anesthetized in 5 mM levamisole in S-basal (0.1 M NaCl, 0.05 M H_2_PO_4_ (pH 6)) and mounted on 2% agarose pads. Worms for DIC and SYTO®12 staining were grown at 20 °C while P_*lim-7*_*ced-1*::GFP worms were grown at 25 °C to obtain a stronger expression of GFP.

### ENU treatment

Germline apoptosis was induced by incubating worms 24 h post the L4 stage in 10 mM N-ethyl-N-nitrosourea (ENU) in S-basal for 60 min, rotating. Worms were pelleted and allowed to recover on fresh NGM or RNAi plates overnight at 20 °C before apoptotic germ cells were quantified by DIC microscopy.

### UV treatment

Germline apoptosis was induced by exposing young adult *rrf-1*;CED-1::GFP worms, approx. 12 h post L4 stage, to UV-C light 254 nm (CL-1000 Ultraviolet crosslinker, UVP) in a doses of 100 J/m^2^ as described^61^. The apoptotic cells in the germline were scored 24 and 48 h after UV treatment.

### Time-lapse microscopy

The engulfment duration of apoptotic cell corpses was monitored by time-lapse microscopy. Four days old worms were anesthetized by 1.5 mM levamisole and subjected to DIC microscopy. The number of corpses was monitored with intervals of 10 min through a period of 120 min. Corpses, which persisted after 120 min, were given the >120 min time point. Corpses, which appeared during the experiment but still persisted at the end of the experiment, were omitted from the data since we did not have their time point of disappearance.

### Immunostaining

Worms were transferred to a poly-l-lysine microscopy slide (VWR, Denmark), the cuticle was punctured with a sharp needle to allow for better exposure of the germline. A coverslip was gently placed on top and freeze-cracking was performed by incubation at −80 °C followed by removal of the coverslip from the frozen slides with a scalpel. The slides were incubated 20 min in ice cold methanol, washed in PBS, and then blocked 2 h in 2% (w/v) milk-PBS. Worms were encircled with a PAP pen (ThermoFischer, Denmark) before incubation with primary antibody against nuclear pore complex (mab414, Covance) 1:1000 dilution in 2% milk-PBS overnight. After incubation, the slides were washed 2 times in PBS and incubated 2 h with goat anti-mouse alexa546 conjugated antibody (ThermoFischer, Denmark) in 2% milk-PBS. The slides were washed 3 times in PBS, fixed 10 min in 2% PFA, and mounted with Vectashield antifade mounting media (Vector Laboratories). Confocal microscopy for co-localization of DLC-1::GFP and nuclear membrane was performed using a Zeiss LSM 780 microscope equipped with a Zeiss AxioCam MRm camera. The ZEN Imaging Software (Zeiss) was used to process the pictures.

### Fluorescence microscopy

Analysis of DLC-1::GFP, DHC-1::GFP, and CED-4::GFP localization was performed using a Zeiss Axiophot microscope equipped with an Andor Zyla 4.2 sCMOS camera.

### Analysis of DHC-1::GFP and CED-4::GFP expression

Fluorescence and DIC microscopy images were loaded into a custom built “NematodeAnalyzer”, a MATLAB program (MATLAB R2014b, Mathworks, Natick, MA, USA). Cells were manually marked on the fluorescence images using a circular ROI. Circular masks, one pixel wide, were generated systematically from the center of the marked ROI and 40 pixels radially outwards. The mean signal intensity of the fluorescence images was calculated for each mask and the maximal value corresponding to the hyperintense signal along the cellular periphery was subtracted by the minimal value corresponding to the background signal. The resulting difference was calculated for all the germ cells using three independent tries, all displaying the same tendency.

### Statistical methods

In all experiments, a Student’s *t*-test was used to calculate the indicated *p* values.

## Electronic supplementary material

Figure S1

Figure S2

Figure S3

Figure S4

Figure S5

Figure S6

Figure S7

Figure S8

Supplemental Materials and Methods
