# Supplementary material for: Dynein links engulfment and execution of apoptosis via CED-4/Apaf1 in C. elegans
Source: Cell Death Dis. 2018 Sep 27;9(10):1012. doi: 10.1038/s41419-018-1067-y (PMC6160458; doi:10.1038/s41419-018-1067-y)
Supplement: Supplementary file 1 — Figure S1 [file 41419_2018_1067_MOESM1_ESM.pdf]

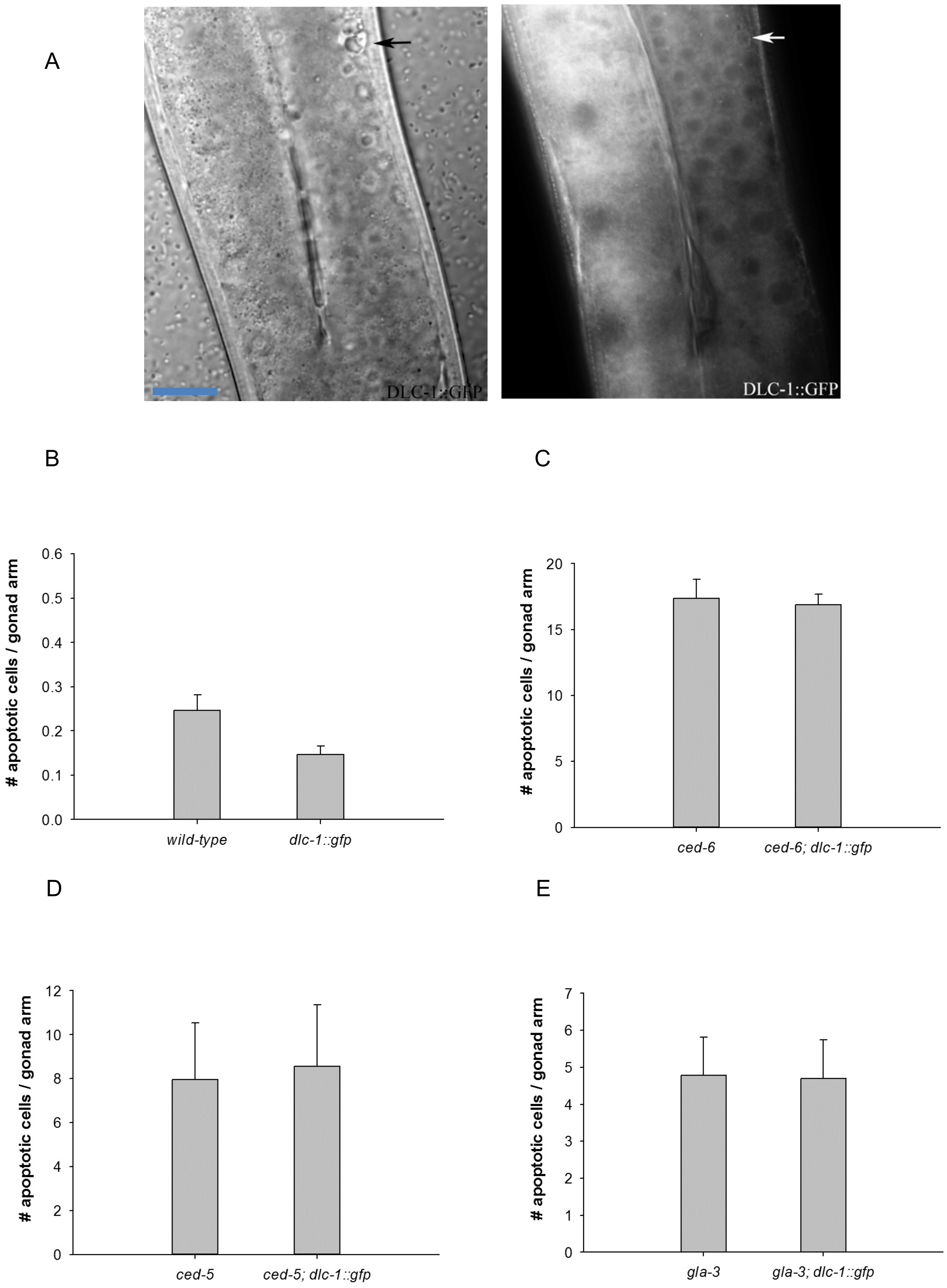
A
DLC-1::GFP
DLC-1::GFP
B
apoptotic cells / gonad arm
0.0
0.1
0.2
0.3
0.4
0.5
0.6
wild-type
dlc-1::gfp
C
apoptotic cells / gonad arm
0
5
10
15
20
ced-6
ced-6; dlc-1::gfp
D
apoptotic cells / gonad arm
0
2
4
6
8
10
12
ced-5
ced-5; dlc-1::gfp
E
apoptotic cells / gonad arm
0
1
2
3
4
5
6
7
gla-3
gla-3; dlc-1::gfp

F

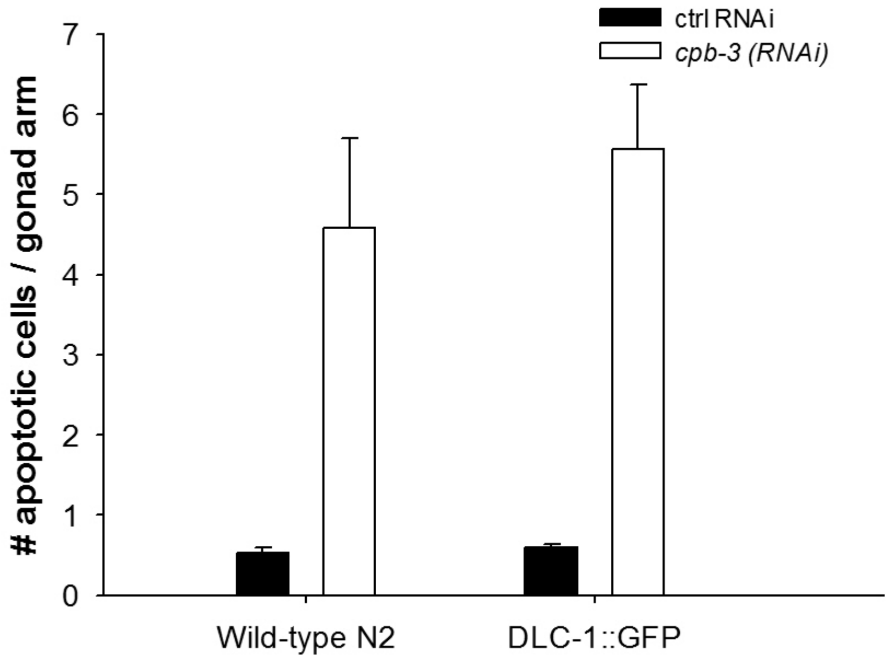

Figure S1. **A**. DLC-1::GFP marks apoptotic corpses (arrow) in wild-type worms. DLC-1::GFP is found evenly distributed at low levels in the germline also on healthy germ cells. Expression of DLC-1::GFP does not significantly alter the level of apoptosis or the morphology of the germ line in wild-type worms (**B**), *ced-6(tm1826)* mutants (**C**), *ced-5(tm1950)* mutants (**D**), *gla-3(ok2684)*mutants (**E**) or animals treated with RNAi against *cbp-3* (**F**). Bars represent mean ± SD of independent experiments for (B) n =2 and for (C), (D), (E) and (F) n = 3.
