# Supplementary figures and images for: Dynein links engulfment and execution of apoptosis via CED-4/Apaf1 in C. elegans
Source: Cell Death Dis. 2018 Sep 27;9(10):1012. doi: 10.1038/s41419-018-1067-y (PMC6160458; doi:10.1038/s41419-018-1067-y)

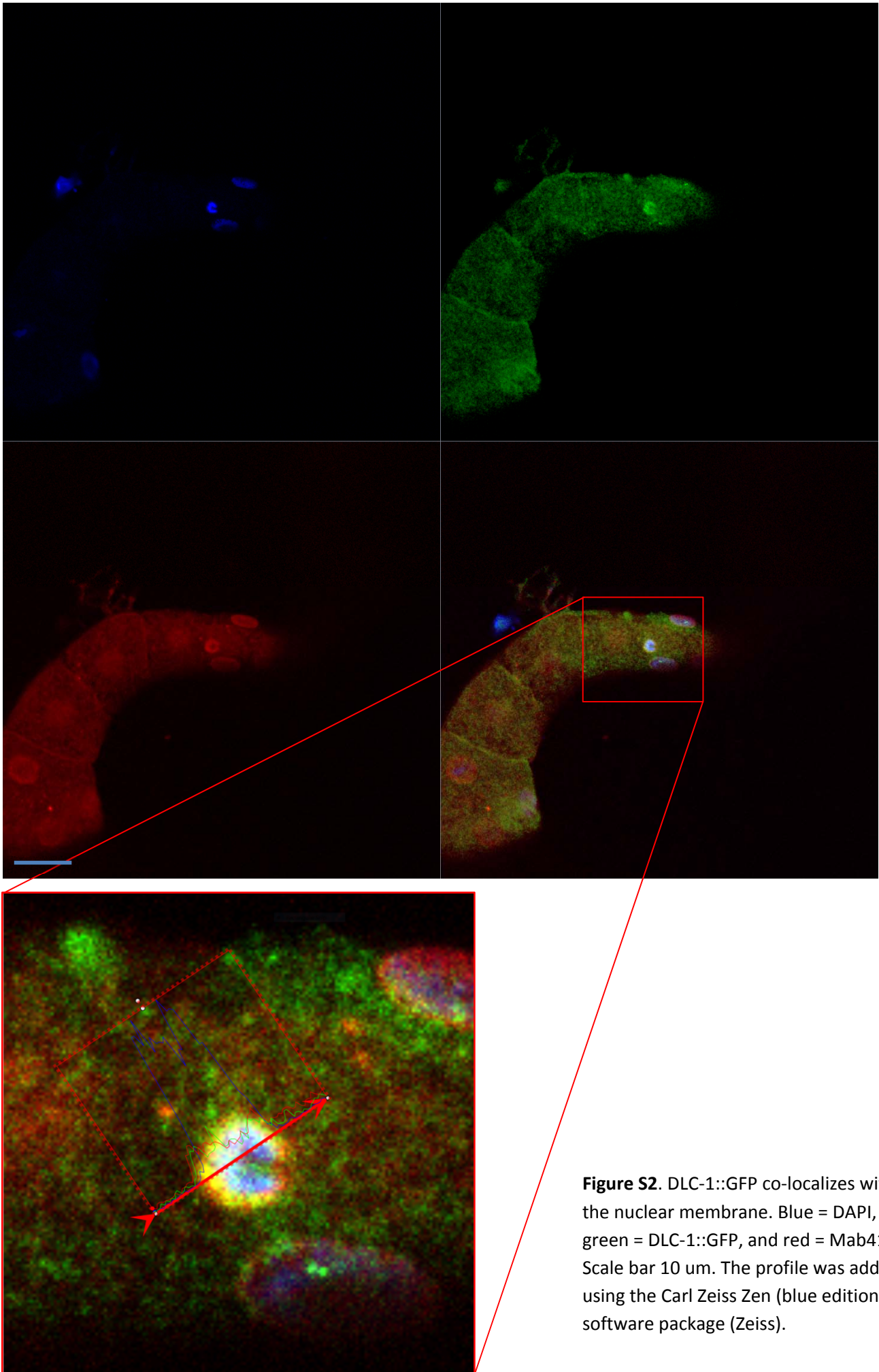

Supplement: Supplementary file 2 — Figure S2 [file 41419_2018_1067_MOESM2_ESM.pdf]
