# Supplementary material for: Dynein links engulfment and execution of apoptosis via CED-4/Apaf1 in C. elegans
Source: Cell Death Dis. 2018 Sep 27;9(10):1012. doi: 10.1038/s41419-018-1067-y (PMC6160458; doi:10.1038/s41419-018-1067-y)
Supplement: Supplementary file 3 — Figure S3 [file 41419_2018_1067_MOESM3_ESM.pdf]

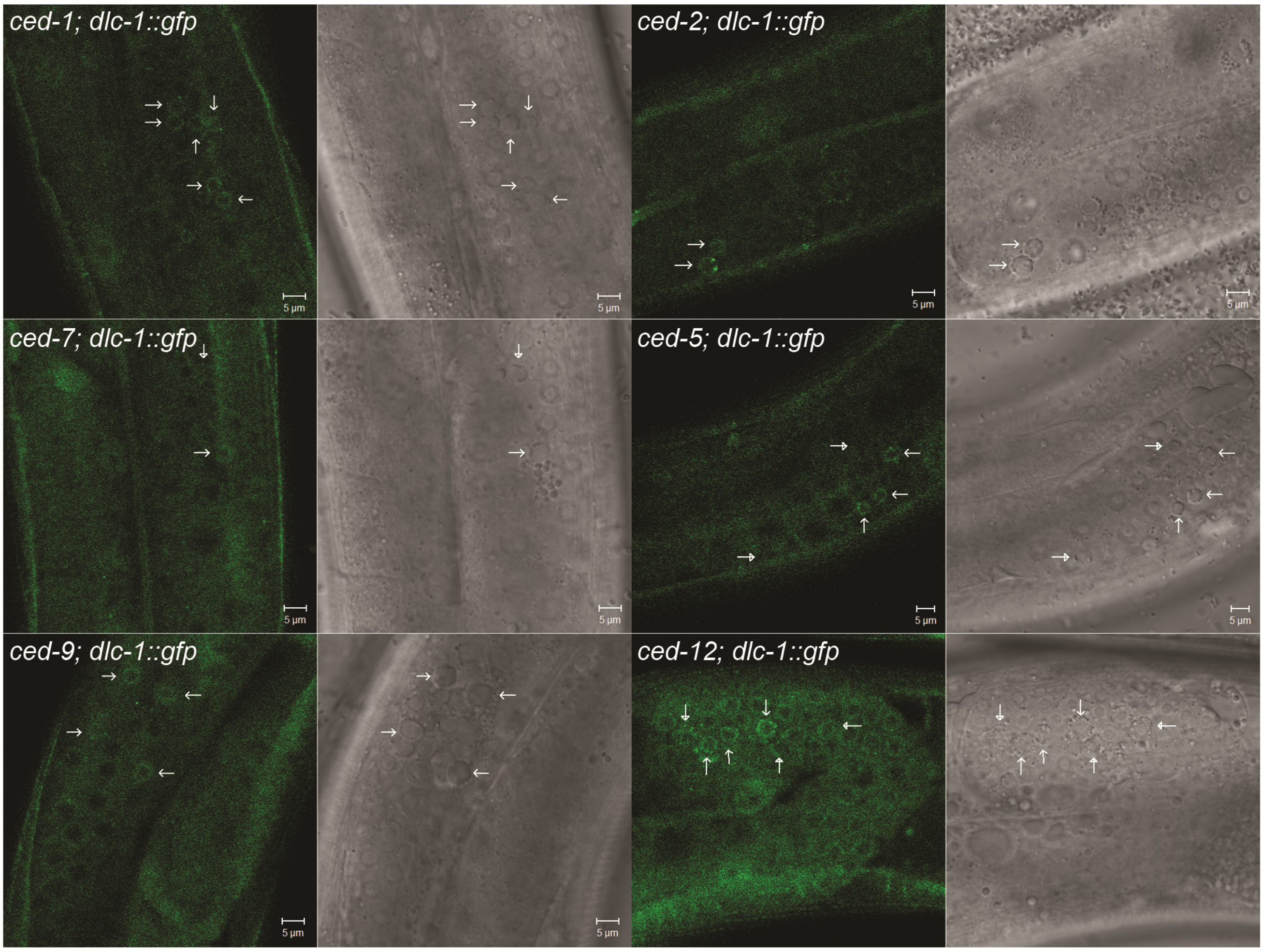

Figure S3. DLC-1::GPF localizes to apoptotic germ cells (white arrows) in mutants of *ced-1(e1735), ced-7(n1996), ced-2(n1994), ced-5(tm1950), ced-12(n3261)* and *ced-9(n1936)*.
