# Supplementary material for: Dynein links engulfment and execution of apoptosis via CED-4/Apaf1 in C. elegans
Source: Cell Death Dis. 2018 Sep 27;9(10):1012. doi: 10.1038/s41419-018-1067-y (PMC6160458; doi:10.1038/s41419-018-1067-y)
Supplement: Supplementary file 4 — Figure S4 [file 41419_2018_1067_MOESM4_ESM.pdf]

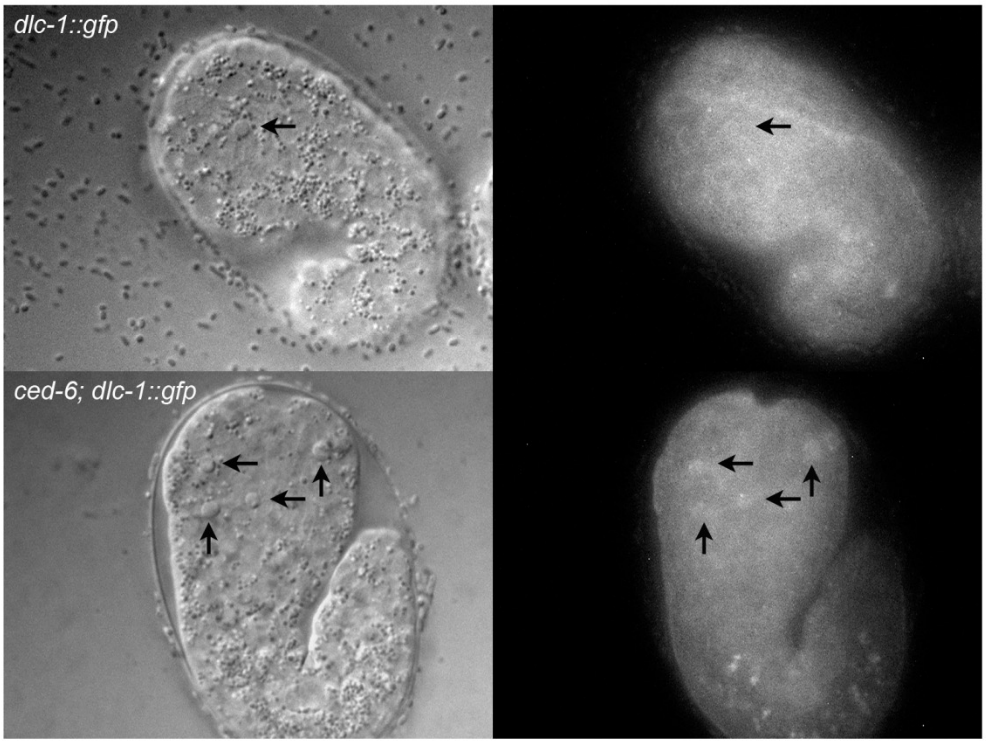

Figure S4. Comma staged embryos of *dlc-1::gfp* and *ced-6(tm1826); dlc-1::gfp*. 100X Magnification. The embryos are approximately 50 um long. DLC-1::GFP does not localize specifically to the cell boundary of DIC positive apoptotic cells.
