# Supplementary material for: Dynein links engulfment and execution of apoptosis via CED-4/Apaf1 in C. elegans
Source: Cell Death Dis. 2018 Sep 27;9(10):1012. doi: 10.1038/s41419-018-1067-y (PMC6160458; doi:10.1038/s41419-018-1067-y)
Supplement: Supplementary file 5 — Figure S5 [file 41419_2018_1067_MOESM5_ESM.pdf]

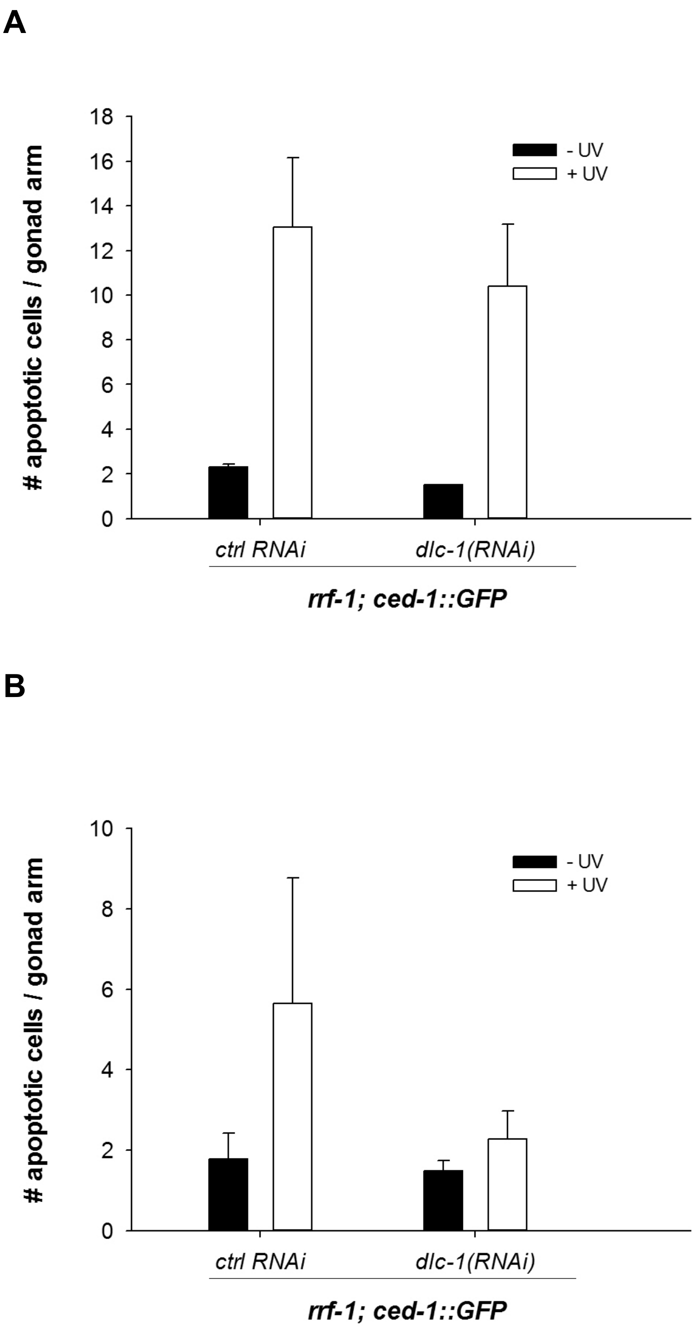

Figure S5. *dlc-1* RNAi does not suppress UV induced apoptosis. Apoptotic corpses were quantified as CED-1::GFP positive cells 24 h (**A**) and 48 h (**B**) after worms were exposed to UV (100 J/m^2). Bars represent mean ± SD of three independent experiments.
