# Supplementary material for: Dynein links engulfment and execution of apoptosis via CED-4/Apaf1 in C. elegans
Source: Cell Death Dis. 2018 Sep 27;9(10):1012. doi: 10.1038/s41419-018-1067-y (PMC6160458; doi:10.1038/s41419-018-1067-y)
Supplement: Supplementary file 6 — Figure S6 [file 41419_2018_1067_MOESM6_ESM.pdf]

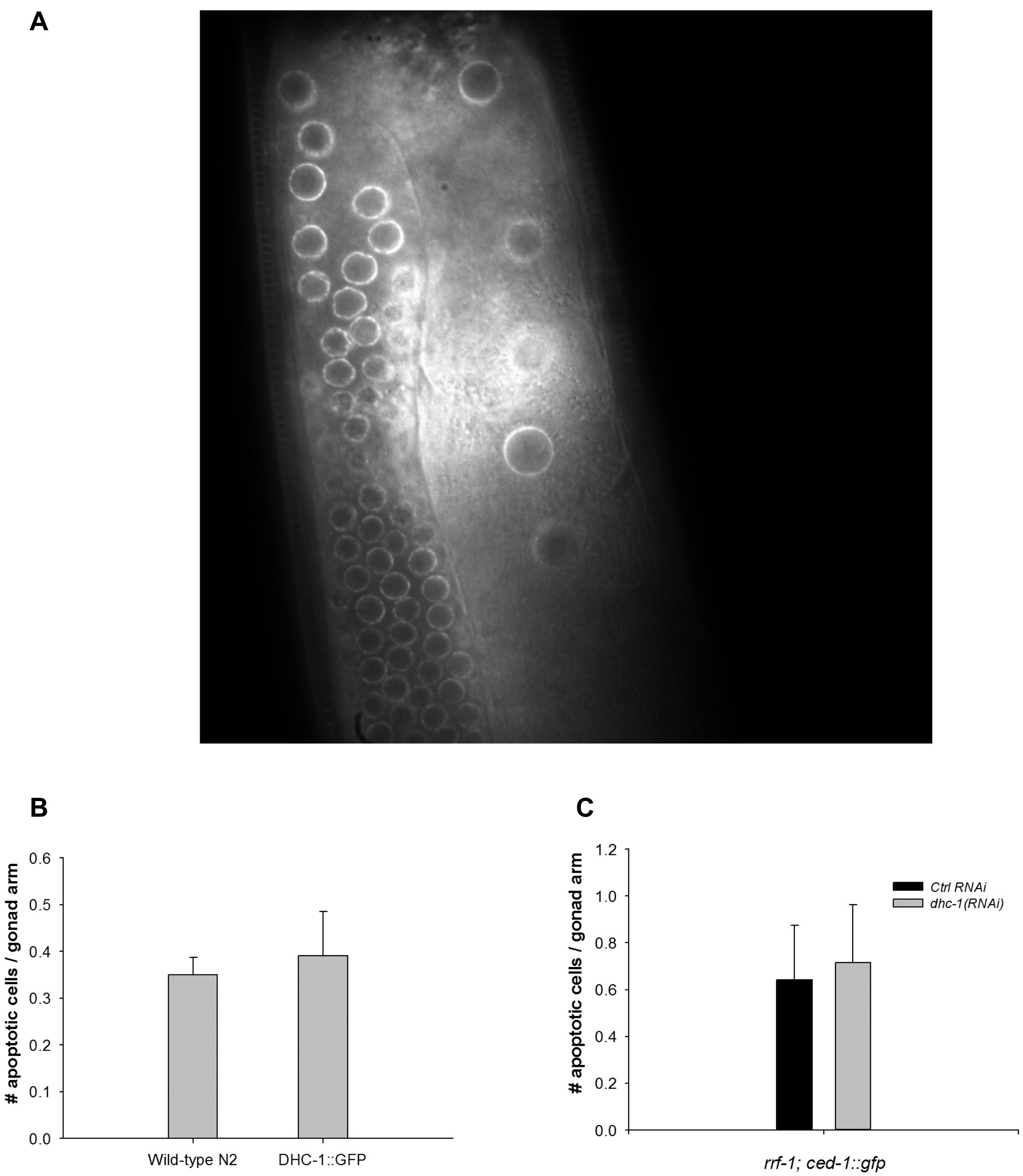

Figure S6. **A**. DHC-1::GFP localizes to the nuclear membrane of germ cells and oocytes. **B**. Expression of DHC-1::GFP does not alter germ line apoptosis or morphology. **C**. RNAi against *dhc-1* does not reduce the number of apoptotic corpses in worms with wild-type engulfment. Bars represent mean ± SD of three independent experiments.
