# Supplementary material for: Dynein links engulfment and execution of apoptosis via CED-4/Apaf1 in C. elegans
Source: Cell Death Dis. 2018 Sep 27;9(10):1012. doi: 10.1038/s41419-018-1067-y (PMC6160458; doi:10.1038/s41419-018-1067-y)
Supplement: Supplementary file 7 — Figure S7 [file 41419_2018_1067_MOESM7_ESM.pdf]

A

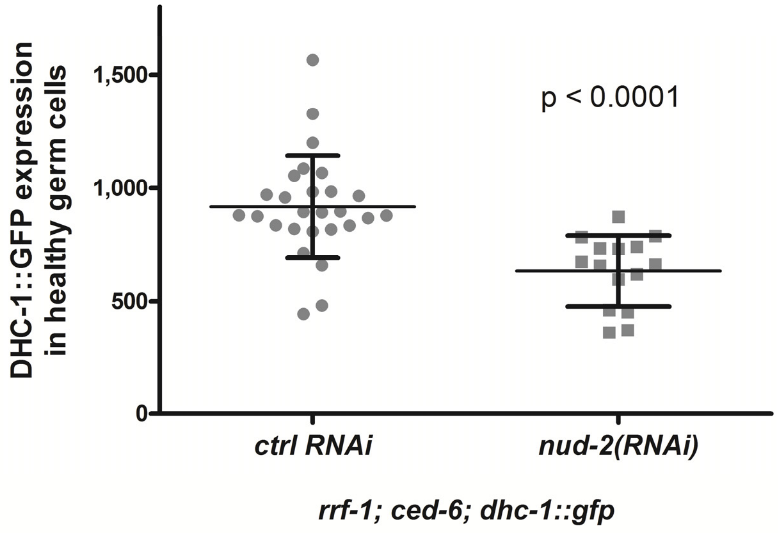
DHC-1::GFP expression in healthy germ cells
1,500
1,000
500
0
p < 0.0001
ctrl RNAi
nud-2(RNAi)
rrf-1; ced-6; dhc-1::gfp

B

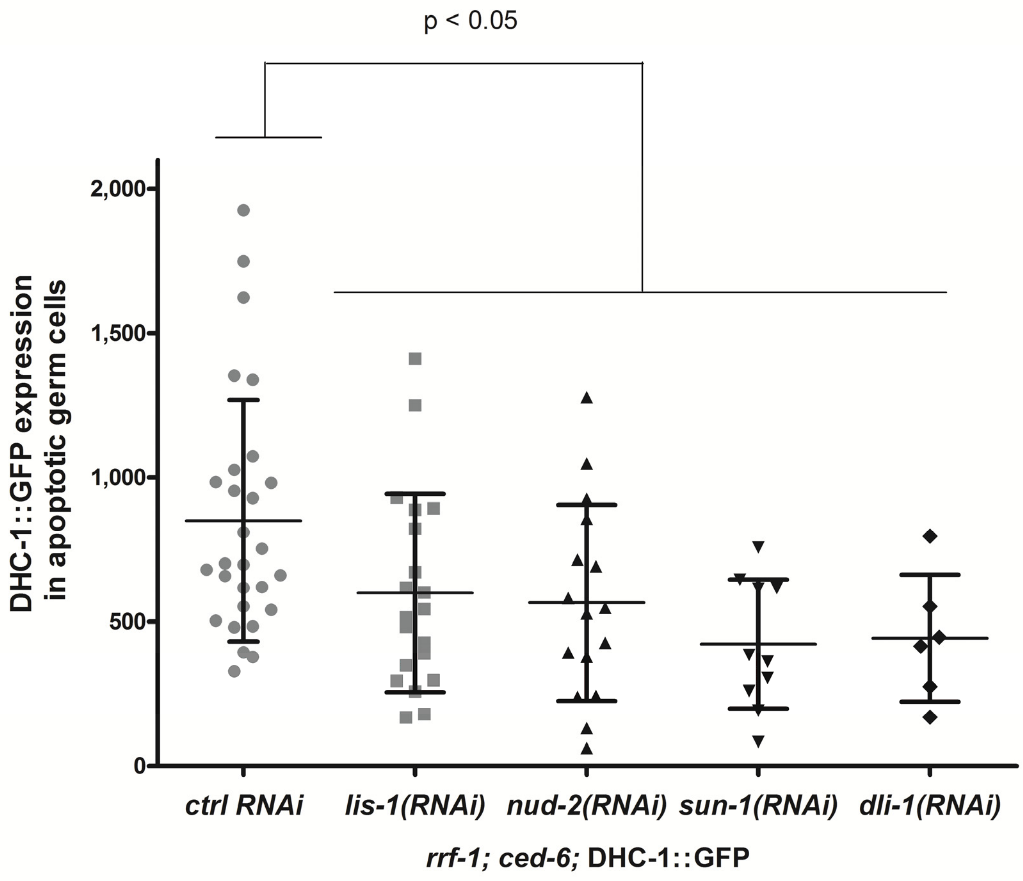
p < 0.05
DHC-1::GFP expression in apoptotic germ cells
2,000
1,500
1,000
500
0
ctrl RNAi
lis-1(RNAi)
nud-2(RNAi)
sun-1(RNAi)
dli-1(RNAi)
rrf-1; ced-6; DHC-1::GFP

C

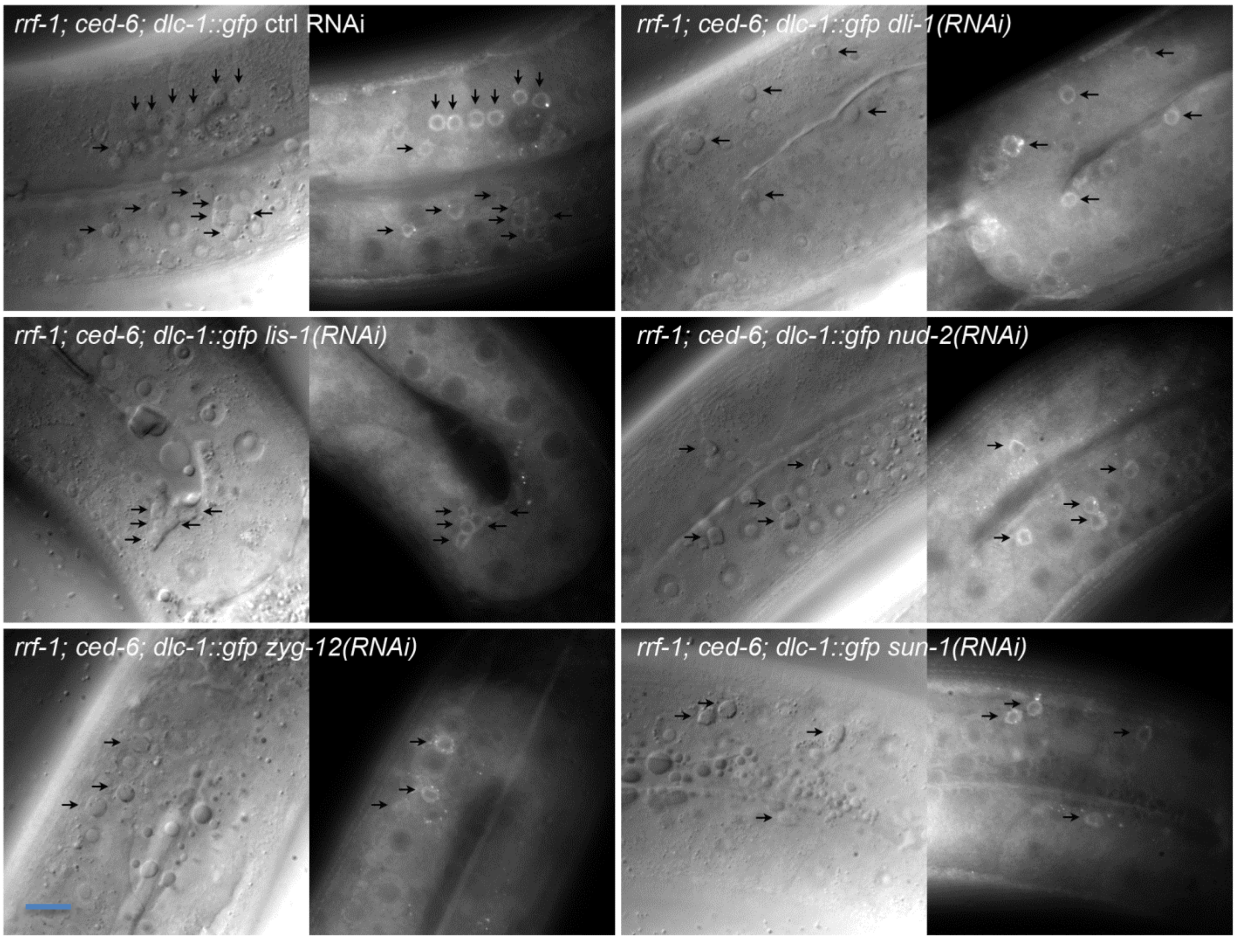

Figure S7. A. Quantification of DHC-1::GFP on healthy germ cells following RNAi of *nud-2*. Due to the low GFP intensity a baseline subtraction based on asymmetric least squares smoothing was performed using OriginPro 2018b (OriginLab Corporation) after the analysis with NematodeAnalyzer. **B**. Quantification of DHC-1::GFP on apoptotic germ cells following RNAi against *lis-1*, *nud-2*, *sun-1*, *dli-1*. Note, only GFP positive cells were quantified. **C**. DLC-1::GFP localization to apoptotic germ cells is not affected by RNAi against *lis-1, zyg-12, nud-2, dli-1* and *sun-1.* Scale bar 10 um.
