# Supplementary material for: Dynein links engulfment and execution of apoptosis via CED-4/Apaf1 in C. elegans
Source: Cell Death Dis. 2018 Sep 27;9(10):1012. doi: 10.1038/s41419-018-1067-y (PMC6160458; doi:10.1038/s41419-018-1067-y)
Supplement: Supplementary file 8 — Figure S8 [file 41419_2018_1067_MOESM8_ESM.pdf]

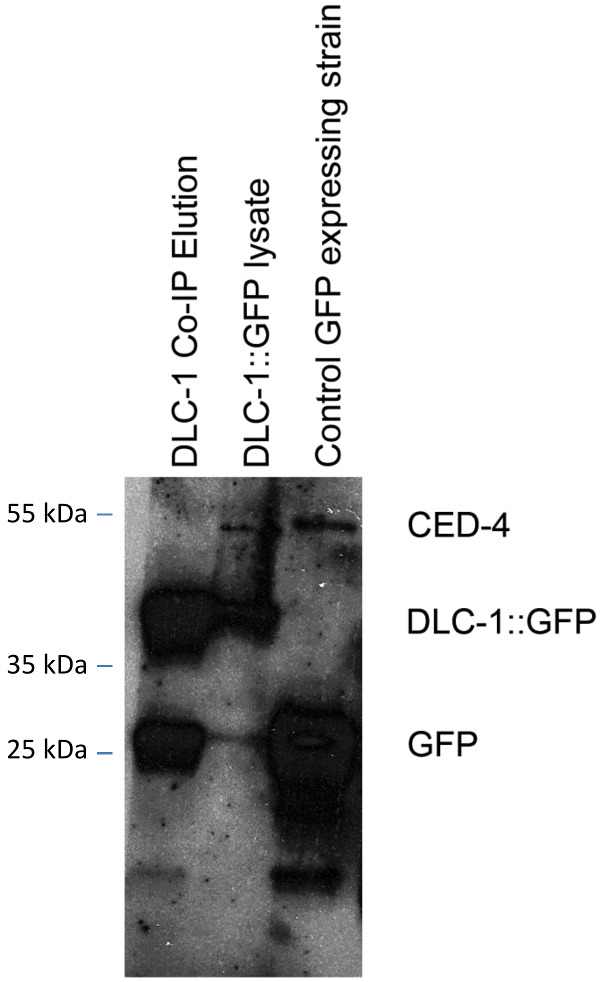

Figure S8. Co-IP of DLC-1::GFP using GFP-Trap® did not pull down CED-4. CED-4 was present in the lysate before binding to the GFP-Trap® beads and in a control strain expressing GFP (VC592). DLC-1::GFP was enriched after elution from the GFP-Trap® beads but no CED-4 was detected. The Thermo Scientific PageRuler Plus Prestained Protein Ladder was used.
