# Supplementary material for: Dynein links engulfment and execution of apoptosis via CED-4/Apaf1 in C. elegans
Source: Cell Death Dis. 2018 Sep 27;9(10):1012. doi: 10.1038/s41419-018-1067-y (PMC6160458; doi:10.1038/s41419-018-1067-y)
Supplement: Supplementary file 9 — Supplemental Materials and Methods [file 41419_2018_1067_MOESM9_ESM.pdf]

Supplemental material

Methods

Co-immuonoprecipitation (CoIP) with GFP-Trap®-MA

CoIP followed by mass spectrometry was performed to see if DLC-1 interacts directly with CED-4. The VC592 mutant strain was used as a positive control for GFP-trapping as it contains free GFP expressed in the pharynx.

DLC-1::GFP worms were synchronized by using Hypo treatment in a larger scale and grown on large plates until adulthood. The worms were harvested, before too many eggs hatched, in a 15 mL tube with S-basal and washed until the bacteria co-transferred, was removed. The worms were gently pelleted and 500 µL were transferred to an Eppendorf tube and snap-freezed in liquid N_2. 500 µL cold GFP-Trap lysis buffer supplemented with 25 µL complete protease inhibitor (Roche) was added to the worms thawed on ice. The worms were grinded in a pre-cooled mortar while pouring liquid N_2. The powder was transferred to a 15 mL tube and 700 µL dilution buffer containing protease inhibitor added. Afterwards, the sample lysates were homogenized (10 strokes) in a Wheaton stainless steel tissue grinder and transferred to a pre-chilled Eppendorf tube. The protein concentration was measured by Nanodrop. The GFP-Trap®-MA beads were vortexed and 25 µL of the bead slurry were transferred into 500 µL ice-cold dilution buffer. The beads were quickly spun and then magnetically separated until the supernatant was clear. The supernatant was discarded and washed twice. The lysates were added to the GFP-Trap®-MA beads and tumbled end-over-end for 1 hour at 4°C. The beads were magnetically separated and the supernatant was discarded. The GFP-Trap®_MA beads were resuspended in 500 µL dilution buffer and washed twice. The proteins bound to the GFP-Trap, were eluted by adding 50 µL elution buffer during constant mixing for 30 sec followed by magnetic separation. Finally, the supernatant were transferred to a new Eppendorf tube containing 5 µL 1M Tris base pH 10.4 for neutralization.

Protein precipitation and tryptic digest

Following elution from CoIP with GFP trap GFP-Trap®-MA, samples from control strain VC592 and DLC-1::GFP tagged worms were processed as follows: Protein concentrations were estimated by measuring absorbance at 280 nm using a NanoDrop 1000 spectrophotometer (Thermo Scientific, Waltham, MA). Proteins (a sample volume corresponding to 4 µg protein/sample) were precipitated in

ice-cold acetone overnight. The precipitated protein pellets were resuspended in 20 µl 50 mM ammonium bicarbonate, pH 8.5. Cysteine residues were reduced in 1 mM dithiotreitol, incubated at 56° C for 1 h, and blocked in 5.5 mM iodoacetamide at 22° C for 30 min in the dark. Proteins were digested overnight at 37° C with 1:50 w/w trypsin. The final peptide samples were dried in a vacuum centrifuge.

LC–MS/MS Sample Analysis

Dry peptide samples were resuspended in 2% acetonitrile and 0.1% formic acid. LC–MS/MS analysis was performed using an UltiMate 3000 UPLC system (Thermo Scientific) coupled online to a Q Exactive Plus mass spectrometer (Thermo Scientific). Peptides (0.5 µg) was separated on a 50 cm C18 Acclaim PepMap100 analytical column (Thermo Scientific) with 96% solvent A (0.1% formic acid) and 4% solvent B (0.1% FA in acetonitrile), which was increased to 30% solvent B on a 230 min ramp gradient at a constant flow rate of 300 nL/min. Eluting peptides were introduced directly into the mass spectrometer by a picotip emitter for electrospray ionization (New Objective, Woburn, MA). The mass spectrometer was operated in positive mode using a data-dependent acquisition method. A full MS scan in the mass range of m/z 400 to 1200 was acquired at a resolution of 70,000. In each cycle, the mass spectrometer would trigger up to 12 MS/MS acquisitions of eluting ions based on highest signal intensity for fragmentation. The MS/MS scans were acquired with a dynamic mass range at a resolution of 17,500. The precursor ions were isolated using a quadrupole isolation window of 1.6 m/z and fragmented using high-energy collision with a normalized collision energy of 27. Fragmented ions were dynamically added to an exclusion list for 30 seconds.

Data processing

Protein identification was performed using Proteome Discoverer (Thermo Scientific) by searching the raw spectral data files against a Uniprot search database containing all reviewed *C. elegans* proteins (downloaded November 11, 2016). Proteome Discoverer relies on an in-house sequence database search program, in this case Mascot (Matrix Science, Boston, MA). All standard settings were employed with carbamidomethyl (C) as a static modification and deamidation (NQ) and oxidation (M) as variable modifications. All proteins were reported with $<1\%$ false discovery rate (FDR) to ensure high-confidence protein identifications.

Supplementary Results

Accession number	Description	Score	Coverage	# Peptides
P47991	60S ribosomal protein L6 OS=Caenorhabditis elegans GN=rpl-6 PE=1 SV=1 - [RL6_CAEEL]	208.04	16.59	4
Q9U6Y5	SWISS-PROT:Q9U6Y5 Green fluorescent protein (GFP-Cter-HisTag)	181.40	42.97	5
P53013	Elongation factor 1-alpha OS=Caenorhabditis elegans GN=eft-3 PE=3 SV=1 - [EF1A_CAEEL]	160.14	23.54	7
P0CG71	Polyubiquitin-A OS=Caenorhabditis elegans GN=ubq-1 PE=3 SV=1 - [UBIQ1_CAEEL]	128.85	32.82	2
P06125	Vitellogenin-5 OS=Caenorhabditis elegans GN=vit-5 PE=2 SV=2 - [VIT5_CAEEL]	127.59	8.23	10
G8JY38	VITellogenin structural genes (Yolk protein genes) OS=Caenorhabditis elegans GN=vit-2 PE=1 SV=1 - [G8JY38_CAEEL]	117.67	8.66	8
Q6A8K1	Actin-4 OS=Caenorhabditis elegans GN=act-4 PE=1 SV=1 - [Q6A8K1_CAEEL]	116.65	17.68	3
P55155	Vitellogenin-1 OS=Caenorhabditis elegans GN=vit-1 PE=1 SV=2 - [VIT1_CAEEL]	106.76	4.27	5
O45946	60S ribosomal protein L18 OS=Caenorhabditis elegans GN=rpl-18 PE=3 SV=1 - [RL18_CAEEL]	93.26	15.96	2
C6KRN1-2	Isoform a of Suppressor of aph-1 OS=Caenorhabditis elegans GN=sao-1 - [SAO1_CAEEL]	85.73	5.80	1
P18948-2	Isoform a of Vitellogenin-6 OS=Caenorhabditis elegans GN=vit-6 - [VIT6_CAEEL]	85.41	8.00	8
P18947	Vitellogenin-4 OS=Caenorhabditis elegans GN=vit-4 PE=1 SV=3 - [VIT4_CAEEL]	83.06	8.42	10
O44480	60S ribosomal protein L18a OS=Caenorhabditis elegans GN=rpl-20 PE=3 SV=2 - [RL18A_CAEEL]	78.79	7.78	1
P09446	Heat shock 70 kDa protein A OS=Caenorhabditis elegans GN=hsp-1 PE=1 SV=2 - [HSP7A_CAEEL]	75.90	10.00	4
Q9N4I4	60S ribosomal protein L10a OS=Caenorhabditis elegans GN=rpl-10a PE=3 SV=1 - [RL10A_CAEEL]	60.66	11.57	2
Q93572	60S acidic ribosomal protein P0 OS=Caenorhabditis elegans GN=rpa-0 PE=1 SV=3 - [RLA0_CAEEL]	60.35	10.90	2
Q9BL19	60S ribosomal protein L17 OS=Caenorhabditis elegans GN=rpl-17 PE=3 SV=1 - [RL17_CAEEL]	57.09	5.35	1
P91913	60S acidic ribosomal protein P1 OS=Caenorhabditis elegans GN=rla-1 PE=3 SV=2 - [RLA1_CAEEL]	53.36	45.95	2
P49181	60S ribosomal protein L36 OS=Caenorhabditis elegans GN=rpl-36 PE=1 SV=3 - [RL36_CAEEL]	52.30	10.58	1
P49405	60S ribosomal protein L5 OS=Caenorhabditis elegans GN=rpl-5 PE=3 SV=1 - [RL5_CAEEL]	50.49	16.38	4
O45713	Uncharacterized protein OS=Caenorhabditis elegans GN=CELE_R09B3.3 PE=1 SV=1 - [O45713_CAEEL]	45.94	32.94	2
O01802	60S ribosomal protein L7 OS=Caenorhabditis elegans GN=rpl-7 PE=3 SV=1 - [RL7_CAEEL]	45.78	9.84	2
Q9XVE9	Ribosomal Protein, Large subunit OS=Caenorhabditis elegans GN=rpl-14 PE=1 SV=1 - [Q9XVE9_CAEEL]	44.80	17.78	2
Q18688	Heat shock protein 90 OS=Caenorhabditis elegans GN=daf-21 PE=1 SV=1 - [HSP90_CAEEL]	42.10	1.99	1
O01692	40S ribosomal protein S17 OS=Caenorhabditis elegans GN=rps-17 PE=3 SV=2 - [RS17_CAEEL]	36.06	12.31	1
Q23312	40S ribosomal protein S7 OS=Caenorhabditis elegans GN=rps-7 PE=3 SV=1 - [RS7_CAEEL]	35.88	18.04	2
P29691	Elongation factor 2 OS=Caenorhabditis elegans GN=eef-2 PE=2 SV=4 - [EF2_CAEEL]	35.69	1.88	1
I2HAJ2	Ribosomal Protein, Large subunit OS=Caenorhabditis elegans GN=rpl-30 PE=1 SV=1 - [I2HAJ2_CAEEL]	33.53	12.77	1
G5EDD4	Tubulin alpha chain OS=Caenorhabditis elegans GN=tba-4 PE=1 SV=1 - [G5EDD4_CAEEL]	31.94	3.35	1
Q21215	Guanine nucleotide-binding protein subunit beta-2-like 1 OS=Caenorhabditis elegans GN=rack-1 PE=1 SV=3 - [GBLP_CAEEL]	31.89	7.69	1
G5EEI4	ASpartyl Protease OS=Caenorhabditis elegans GN=asp-1 PE=1 SV=1 - [G5EEI4_CAEEL]	31.73	4.29	1
P48158	60S ribosomal protein L23 OS=Caenorhabditis elegans GN=rpl-23 PE=3 SV=1 - [RL23_CAEEL]	30.68	7.86	1
P27798	Calreticulin OS=Caenorhabditis elegans GN=crt-1 PE=3 SV=1 - [CALR_CAEEL]	30.33	7.34	1
P54412-2	Isoform b of Probable elongation factor 1-gamma OS=Caenorhabditis elegans GN=eef-1G - [EF1G_CAEEL]	29.57	5.09	1
P91374	60S ribosomal protein L15 OS=Caenorhabditis elegans GN=rpl-15 PE=3 SV=1 - [RL15_CAEEL]	29.34	12.75	1
I2HAF8	40S ribosomal protein S6 OS=Caenorhabditis elegans GN=rps-6 PE=1 SV=1 - [I2HAF8_CAEEL]	29.21	7.51	1
O45499	40S ribosomal protein S26 OS=Caenorhabditis elegans GN=rps-26 PE=3 SV=1 - [RS26_CAEEL]	29.03	12.82	1
Q9BKU6	Uncharacterized protein OS=Caenorhabditis elegans GN=CELE_Y37E3.8 PE=1 SV=1 - [Q9BKU6_CAEEL]	28.91	23.86	2
Q19877	40S ribosomal protein S23 OS=Caenorhabditis elegans GN=rps-23 PE=3 SV=1 - [RS23_CAEEL]	28.09	7.69	1
P27604	Adenosylhomocysteinase OS=Caenorhabditis elegans GN=ahcy-1 PE=3 SV=1 - [SAHH_CAEEL]	27.99	9.38	2
P91128	60S ribosomal protein L13 OS=Caenorhabditis elegans GN=rpl-13 PE=3 SV=1 - [RL13_CAEEL]	27.43	14.49	2
O62337	Uncharacterized protein OS=Caenorhabditis elegans GN=CELE_R06C1.4 PE=1 SV=1 - [O62337_CAEEL]	26.12	17.86	1
Q95Y04	40S ribosomal protein S28 OS=Caenorhabditis elegans GN=rps-28 PE=3 SV=1 - [RS28_CAEEL]	25.36	41.54	2
Q8IG31	PeRoxireDoXin OS=Caenorhabditis elegans GN=prdx-2 PE=1 SV=1 - [Q8IG31_CAEEL]	23.85	6.15	1
Q95Y90	60S ribosomal protein L9 OS=Caenorhabditis elegans GN=rpl-9 PE=3 SV=1 - [RL9_CAEEL]	23.15	8.99	1
Q22799	Dynein light chain 1, cytoplasmic OS=Caenorhabditis elegans GN=dlc-1 PE=1 SV=1 - [DYL1_CAEEL]	23.12	13.48	2
P46561	ATP synthase subunit beta, mitochondrial OS=Caenorhabditis elegans GN=atp-2 PE=1 SV=2 - [ATPB_CAEEL]	22.37	3.53	1
O02639	60S ribosomal protein L19 OS=Caenorhabditis elegans GN=rpl-19 PE=3 SV=1 - [RL19_CAEEL]	22.18	8.59	1
Q10454-2	Isoform b of Probable arginine kinase F46H5.3 OS=Caenorhabditis elegans GN=F46H5.3 - [KARG1_CAEEL]	22.14	3.90	1
Q18253	Dipeptidyl peptidase family member 2 OS=Caenorhabditis elegans GN=dpf-2 PE=1 SV=1 - [DPF2_CAEEL]	21.80	1.21	1

Table S1. List of the 49 *C. elegans* proteins (plus the GFP-tag) identified from the sample derived from DLC-1::GFP worms after elution from Co-IP with GFP-Trap®. The four accessions highlighted in green are proteins that are exclusively found in the DLC-1::GFP sample. These are not present in the Co-IP with samples derived from non-protein tagged GFP worms. Score reflects the combined scores of all observed mass spectra that can be matched to amino acid sequences within that protein. A higher score indicates a more confident match. Coverage represents the percentage of the protein that has been mapped by matched peptides, while number of different peptides mapped to the given protein is also shown.
